# Evolutionary Origin of GnIH and NPFF in Chordates: Insights from Novel Amphioxus RFamide Peptides

**DOI:** 10.1371/journal.pone.0100962

**Published:** 2014-07-01

**Authors:** Tomohiro Osugi, Tomoki Okamura, You Lee Son, Makoto Ohkubo, Takayoshi Ubuka, Yasuhisa Henmi, Kazuyoshi Tsutsui

**Affiliations:** 1 Laboratory of Integrative Brain Sciences, Department of Biology, Waseda University, and Center for Medical Life Science of Waseda University, Tokyo, Japan; 2 Aitsu Marine Station, Center for Marine Environmental Studies, Kumamoto University, Kumamoto, Japan; University of Rouen, France, France

## Abstract

Gonadotropin-inhibitory hormone (GnIH) is a newly identified hypothalamic neuropeptide that inhibits pituitary hormone secretion in vertebrates. GnIH has an LPXRFamide (X = L or Q) motif at the C-terminal in representative species of gnathostomes. On the other hand, neuropeptide FF (NPFF), a neuropeptide characterized as a pain-modulatory neuropeptide, in vertebrates has a PQRFamide motif similar to the C-terminal of GnIH, suggesting that GnIH and NPFF have diverged from a common ancestor. Because GnIH and NPFF belong to the RFamide peptide family in vertebrates, protochordate RFamide peptides may provide important insights into the evolutionary origin of GnIH and NPFF. In this study, we identified a novel gene encoding RFamide peptides and two genes of their putative receptors in the amphioxus *Branchiostoma japonicum*. Molecular phylogenetic analysis and synteny analysis indicated that these genes are closely related to the genes of GnIH and NPFF and their receptors of vertebrates. We further identified mature RFamide peptides and their receptors in protochordates. The identified amphioxus RFamide peptides inhibited forskolin induced cAMP signaling in the COS-7 cells with one of the identified amphioxus RFamide peptide receptors expressed. These results indicate that the identified protochordate RFamide peptide gene is a common ancestral form of GnIH and NPFF genes, suggesting that the origin of GnIH and NPFF may date back to the time of the emergence of early chordates. GnIH gene and NPFF gene may have diverged by whole-genome duplication in the course of vertebrate evolution.

## Introduction

Gonadotropin-inhibitory hormone (GnIH) is a newly identified hypothalamic neuropeptide that inhibits pituitary hormone secretion in vertebrates [1]–[4]. The discovery of GnIH has now changed drastically the classical understanding of the regulation of hypothalamic-pituitary-gonadal (HPG) axis in vertebrates [1]–[4]. In addition, a recent study demonstrated that hypothalamic GnIH inhibits socio-sexual behaviors by increasing neuroestrogen synthesis in male birds [5]. After the discovery of GnIH in birds, its orthologous peptides have been identified in a variety of vertebrates, such as primates and mammals [RFamide-related peptides (RFRP)] [6]–[9], amphibians [frog growth hormone-releasing peptide (fGRP) [10], [11], *Rana* RFamide [12], and newt LPXRFamide peptide [13]], and teleosts [goldfish (gf)LPXRFamide peptide] [14]. All the identified GnIH and its orthologs possessed a C-terminal Leu-Pro-Xaa-Arg-Phe-NH_2_ (Xaa  =  Leu or Gln) motif at their C-termini, and thus, they were designated structurally as LPXRFamide (X = L or Q) peptides (LPXRFa peptides). LPXRFa peptides constitute one of the largest groups in the RFamide peptide (RFa peptide) family in vertebrates [2]–[4]. Typically, the LPXRFa peptide precursors in vertebrates encode two to four LPXRFa peptides, such as RFRP-1 and RFRP-3 in primates and mammals [7]–[9]; GnIH, GnIH gene-related peptide (RP)-1 and GnIH-RP-2 in birds [15]–[18]; fGRP, fGRP-RP-1, fGRP-RP-2, and fGRP-RP-3 in amphibians [19]; and LPXRFa-1, LPXRFa-2, and LPXRFa-3 in fish [14], [20], [21] (see Table S1).

From a structural point of view, pain modulatory neuropeptides, such as neuropeptide FF (NPFF) and NPAF, share a C-terminal Pro-Gln-Arg-Phe-NH_2_ motif (PQRFa peptides) [22]. Interestingly, the C-terminal motifs of GnIH and NPFF group peptides showed high sequence similarity, which are considered to be important for the interaction with their receptors (GPR147 and 74) [2]–[4], [22]. The structure of their receptors also showed high sequence similarity. In addition, synteny analyses showed that GnIH gene and NPFF gene are both located near the *HOX* clusters [23], [24]. Because the *HOX* clusters are considered to have duplicated from a common ancestral gene during whole-genome duplication events through vertebrate evolution [25], GnIH gene and NPFF gene may also have diverged through whole-genome duplication in the course of evolution in vertebrates [23], [24]. In our recent studies, the orthologs of GnIH and NPFF genes were identified from the brains of agnathans (lamprey and hagfish), the most ancient lineage of vertebrates [24], [26], [27]. Therefore, the origin of GnIH and NPFF genes may date back before the emergence of early vertebrates.

Amphioxus is considered to be the most basal chordates [28]. The recent genome sequencing analysis using *Branchiostoma floridae* revealed that a single *HOX* cluster is present in contrast to the multiple *HOX* clusters in vertebrates [29]. The single *HOX* cluster of amphioxus may reflect the primitive prevertebrate condition and this character suggests that the common ancestor of chordates may have not experienced the whole-genome duplication [30], [31]. Based on these characters, the amphioxus is considered to be an excellent animal model to investigate the evolutionary origin of GnIH and NPFF genes in chordates. Therefore, in this study we sought to identify novel RFa peptides and their receptors in the amphioxus *Branchiostoma japonicum*.

## Materials and Methods

### Animals

Adult amphioxus (*Branchiostoma japonicum*) were collected in the southern half of the Ariake Sea (Shimabara Bay) in Kumamoto Prefecture, Japan in June 2011. The detailed method of the collection of amphioxus was described in Henmi and Yamaguchi [32]. The experimental protocols were approved by the committee of Waseda University and Kumamoto University, and performed according to the Guide for the Care and Use of Animals prepared by the Waseda University and Kumamoto University.

### Bioinformatics Analyses

The Joint Genome Institute (JGI) genome database of the amphioxus *Branchiostoma floridae* (http://genome.jgi-psf.org/Brafl1/Brafl1.home.html) and Ensembl Genome Browser (http://www.ensembl.org/index.html) was used for the bioinformatics analyses. GENSCAN [33] (http://genes.mit.edu/GENSCAN.html) was used to obtain putative full length cDNA sequences of amphioxus PQRFa peptide gene or its receptor genes. The detail method is described in Materials and Methods S1.

### Molecular Cloning

Based on the nucleotide sequences of the scaffold Bf_V2_187 for amphioxus PQRFa peptide precursor gene, scaffold BF_V2_167 for amphioxus PQRFa-R1 and scaffold BF_V2_95 for amphioxus PQRFa-R2 in the genome database of *Branchiostoma floridae*, we performed molecular cloning of the genes using the tissues of *Branchiostoma japonicum*. First-strand cDNA was synthesized with the oligo(dT)-anchor primer supplied in the 5′/3′ rapid amplification of cDNA ends (RACE) kit (Roche Diagnostics, Basel, Switzerland). The sequence of amphioxus PQRFa peptide precursor cDNA and PQRFa receptor cDNAs were determined as described previously [24], [26], [27]. All primers used in this study are summarized in Table S2. The detail method is described in Materials and Methods S1. The sequence reported in this paper has been deposited in the DDBJ, EMBL, and GenBank database (accession numbers AB863739, AB863740, AB863741 for cDNA sequences of amphioxus PQRFa peptide precursor, amphioxus PQRFa peptide receptor 1and 2).

### Phylogenetic Analysis

Multiple sequence alignments and phylogenetic analyses of the amphioxus PQRFa peptide precursor and vertebrate GnIH and NPFF precursors were performed by using MEGA5 [34]. The phylogenetic tree was constructed by the neighbor-joining method. The data were obtained from 1000 bootstrap replicates to determine the confidence indices within the phylogenetic tree. The bootstrap values were shown as percentage after 1000 replications on the branches. The GenBank accession numbers of the sequences used in the phylogenetic analysis are shown in Tables S3 and S4. The alignments used for the construction of the phylogenetic trees are shown in Figures S1 and S2.

### Peptide Extraction and Immunoaffinity Purification

The tissues of 250 adult amphioxus were used for the peptide extraction as described previously [24], [26], [27]. The affinity chromatography was performed as described previously [26], [27]. The antiserum against PQRFa peptide [26] was conjugated to Protein A Sepharose 4B (Amersham Pharmacia Biotech, Uppsala, Sweden) as an affinity ligand. The tissue extract was applied to the column at 4°C, and the adsorbed materials were eluted with 0.3 M acetic acid containing 0.1% 2-mercaptoethanol. An aliquot of each fraction (1 ml) was analyzed by a dot immunoblot assay with the antiserum against PQRFa peptide according to our previous methods [24], [26], [27]. The detail method is described in Materials and Methods S1.

### HPLC and Structure Determination

The immunoreactive materials obtained by immunoaffinity purification using the antiserum against PQRFa peptide [26] were subjected to a HPLC column (ODS-80TM; Tosoh, Tokyo, Japan) with a linear gradient of 10–50% acetonitrile containing 0.1% trifluoroacetic acid for 100 min at a flow rate of 0.5 ml/min, and the eluted fractions were collected every 2 min. All the fractions were analyzed by the MALDI-TOF MS (AXIMA-CFR plus; Shimadzu) to search the molecular mass values of the amphioxus PQRFa peptides that were predicted from the precursor protein sequence. Tandem mass spectra were acquired on the MALDI-TOF MS in post-source decay mode to confirm the amino acid sequences of amphioxus PQRFa peptides. The theoretical mass values of amphioxus PQRFa peptides and their fragment ions were calculated based on the peptide sequences with C-terminal amidation by Protein Prospector version 5.9.0 (http://prospector.ucsf.edu/prospector/mshome.htm).

### Transient Transfection and Luciferase Assay

COS-7 cells were maintained in Dulbecco's modified Eagle's medium (DMEM; GIBCO/Invitrogen, Carlsbad, CA) supplemented with high glucose (4.5 g/L) containing 10% fetal bovine serum (GIBCO/Invitrogen) and 1% penicillin/streptomycin antibiotics (GIBCO/Invitrogen) in a humidified 5% CO_2_ atmosphere at 37°C. For luciferase assay, COS-7 cells were plated in 24-well plates and grown to 70–80% confluence for 24 h before transfection. Cells were then co-transfected with 200 ng of p3XFLAG-CMV-14 expression vector (Sigma-Aldrich Co., St Louis, MO) containing the amphioxus PQRFa-R1, PQRFa-R2, or empty vector, 200 ng of the pGL4.29[luc2P/CRE/Hygro] (firefly luciferase reporter construct; Promega, Madison, WI) and 5 ng of pRL-null (renilla luciferase reporter construct; Promega) using FuGENE HD Transfection Reagent (Promega) according to the manufacturer's instructions. Cells were starved overnight in serum-free DMEM and then challenged by forskolin (FSK) (Santa Cruz Biotechnology, Santa Cruz, CA) and amphioxus PQRFa peptides (PQRFa-1, -2 and -3) for 6 h. Cell extracts were prepared, and luciferase activity was measured using the dual-luciferase reporter assay system (Promega). The ratio of firefly luciferase activity to renilla luciferase activity was used as the results to coordinate the differences in transfection efficiency among samples. All assays were performed in duplicate and repeated three times. Statistical significance was assessed by Prism statistical software (GraphPad Software Inc., La Jolla, CA). The detail method is described in Materials and Methods S1.

## Results

### Molecular Characterization of Amphioxus PQRFa Peptide Precursor

**Figure 1:**
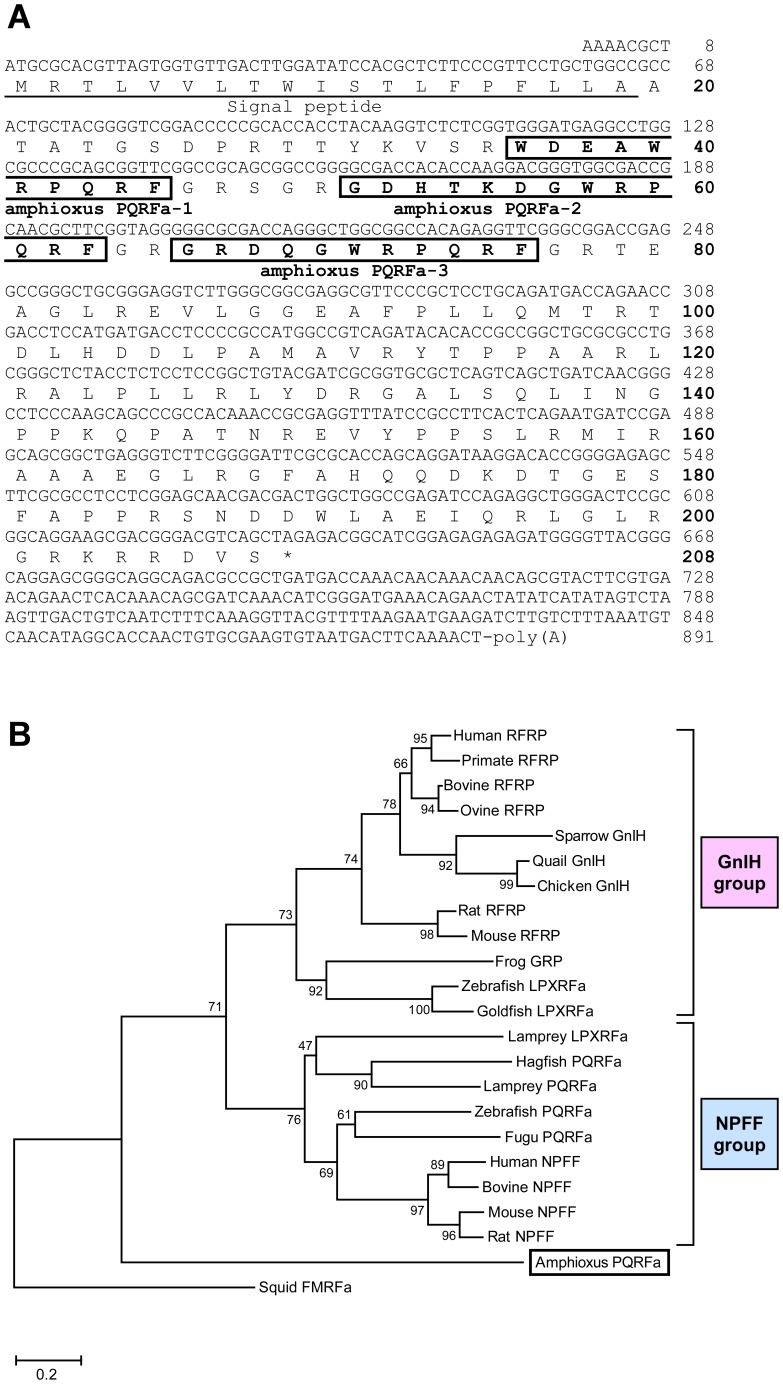
cDNA sequence encoding amphioxus PQRFa peptides and phylogenetic analysis. (*A*) Nucleotide sequence and deduced amino acid sequence of a cDNA encoding putative amphioxus PQRFa-1, amphioxus PQRFa-2, and amphioxus PQRFa-3. The sequences of these putative amphioxus peptides are shown in bold. The signal peptide (19 aa) is underlined. (*B*) Rooted phylogenetic tree of amphioxus PQRFa peptide precursor, identified or putative vertebrate GnIH precursors and NPFF precursors. Numbers on the branches indicate bootstrap percentage after 1000 replications in constructing the tree. The position of amphioxus PQRFa precursor is boxed. Lamprey LPXRFa precursor was included in the NPFF group due to the low amino acid homology to the precursors of GnIH group. Scale bar refers to a phylogenetic distance of 0.1 nucleotide substitutions per site.

We cloned an amphioxus PQRFa peptide precursor cDNA in *Branchiostoma japonicum* based on the nucleotide sequences obtained from the genome database of *Branchiostoma floridae* (Figure S3). Figure 1A shows that the deduced amphioxus PQRFa peptide precursor polypeptide encoded three putative amphioxus PQRFa peptides (amphioxus PQRFa-1, -2 and -3) that included PQRF sequence at their C-termini. The amphioxus PQRFa peptide precursor cDNA was composed of 891 nucleotides containing a short 5′ untranslated region (UTR) of 8 bp, a single open reading frame (ORF) of 624 bp, and a 3′ UTR of 259 bp with a poly(A) tail. The ORF region began with a start codon at position 9 and terminated with a TAG stop codon at position 633. We predicted that the amphioxus PQRFa transcript may be translated from Met^1^ because a hydropathy plot analysis of the precursor showed that the most hydrophobic moiety, which is typical in a signal peptide region, followed Met^1^. The cleavage site of the predicted signal peptide was the Ala^19^–Ala^20^ bond, which is supported by the -3, -1 rule [35]. In this study, a phylogenetic tree was constructed by using neighbor joining method based on amino acid sequences of the amphioxus PQRFa peptide precursor, vertebrate GnIH precursors and vertebrate NPFF precursors. Squid FMRFa precursor was used as an out group. As shown in Figure 1B, the amphioxus PQRFa peptide precursor located before the split of GnIH group and NPFF group.

### Synteny Analysis of Amphioxus PQRFa Peptide Gene Locus

**Figure 2:**
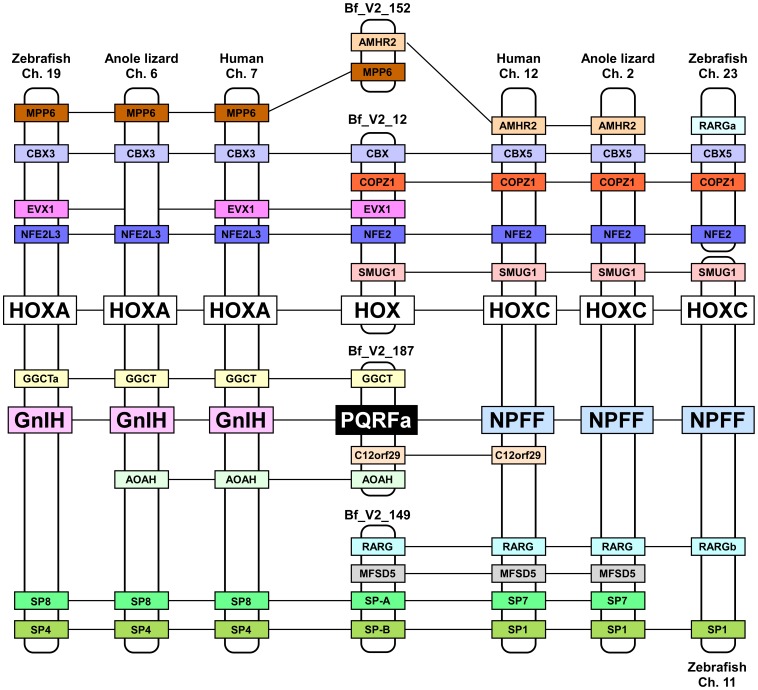
Synteny analysis of the amphioxus PQRFa peptide gene locus. The name of animals and chromosome numbers are shown on the top or bottom of each chromosome or scaffold region. Orthologous genes are linked by horizontal lines. The amphioxus PQRFa peptide gene, vertebrate GnIH genes or vertebrate NPFF genes are shown in black, pink or blue boxes. *HOX* clusters are shown in white boxes. The conserved synteny region exists around the loci of amphioxus PQRFa peptide gene, GnIH gene and NPFF gene.

Synteny analysis showed that the paralogous genes, such as *CBX3* and *CBX5* or *SP1* and *SP4*, or *HOXA* and *HOXC* are located in the conserved synteny region of GnIH and NPFF gene loci in vertebrates (Figure 2). Some orthologous genes seen around GnIH gene or NPFF gene loci are located near the amphioxus PQRFa peptide gene locus in the scaffold Bf_V2_187 (Figure 2). Although the amphioxus genome data are incomplete, many orthologous genes including *HOX* cluster are found in the scaffold Bf_V2_12, Bf_V2_149 or Bf_V2_152 (Figure 2). These data suggest that the synteny region was conserved among the loci around amphioxus PQRFa peptide gene, vertebrate GnIH genes and vertebrate NPFF genes.

### Isolation and Characterization of Endogenous Amphioxus PQRFa Peptides

**Figure 3:**
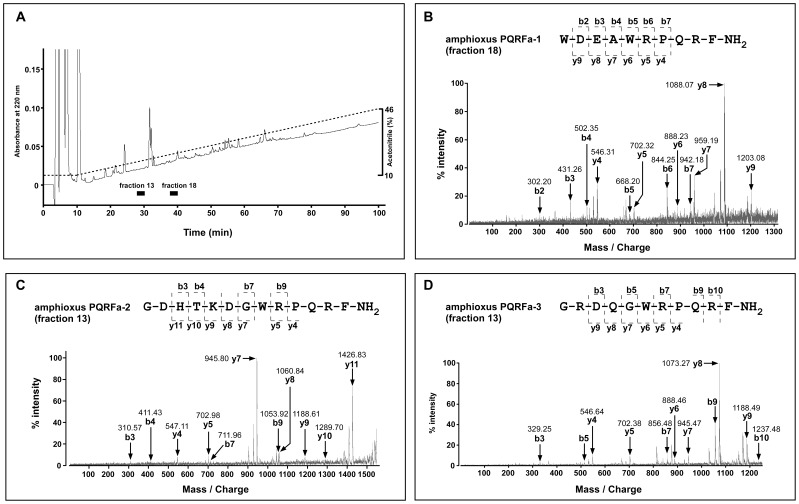
Purification and identification of endogenous amphioxus PQRFa peptides. (*A*) HPLC profile of the retained material obtained by immunoaffinity purification using the antiserum against PQRFa peptide. The immunoreactive substances were eluted in fraction 13 (17–18% acetonitrile) and fraction 18 (21–22% acetonitrile), indicated by the horizontal bars. (*B–D*) Detection of native amphioxus PQRFa peptides by MALDI-TOF MS. Fragmentation patterns of amphioxus PQRFa-1 (*B*), PQRFa-2 (*C*) and PQRFa-3 (*D*) by post-source decay analysis on MALDI-TOF MS. The spectrum shows typical mass values of predicted fragment ions. Observed N-terminal (b) and C-terminal (y) fragmentation ions are assigned in the sequence of each amphioxus PQRFa peptides.

The endogenous amphioxus PQRFa peptides were identified to test the biological activity of these peptides. As shown in Figure 3A, immunopurified material was subjected to reverse-phase HPLC purification with a linear gradient of 10–50% acetonitrile for 100 min. Each fraction was examined by matrix assisted laser desorption/ionization time-of-flight mass spectrometry (MALDI-TOF MS). Molecular ion peaks in the spectrum of the fraction corresponding to the elution times of 28–30 min was observed at 1401.72 m/z ([M+H]^+^) and 1598.67 m/z ([M+H]^+^) (Figure S2A). A molecular ion peak in the fraction corresponding to the elution times of 38–40 was observed at 1389.97 m/z ([M+H]^+^) (Figure S4B). These values were close to the mass numbers of 1401.72 m/z [(M+H)^ +^] (amphioxus PQRFa-3), 1598.79 m/z [(M+H)^ +^] (amphioxus PQRFa-2), and 1389.68 m/z [(M+H)^ +^] (amphioxus PQRFa-1) calculated for the deduced amidated peptide sequence, respectively. By tandem mass spectrometry, the typical fragment ion peaks of each peptide were observed (Figure 3 B–D). The fragment ion peaks of each peptide observed by tandem mass spectrometry were in agreement with the theoretical values. Therefore, the amino acid sequences of each peptide were determined as WDEAWRPQRF-NH_2_ (amphioxus PQRFa-1), GDHTKDGWRPQRF-NH_2_ (amphioxus PQRFa-2) and GRDQGWRPQRF-NH_2_ (amphioxus PQRFa-3). The identified amphioxus PQRFa peptides were compared to vertebrate peptides of the GnIH group and the NPFF group that were previously identified from agnathans to humans (Table S1). The C-terminal six amino acids (WRPQRF) were conserved among amphioxus PQRFa peptides (Table S1). The C-terminal PQRFa motif was identical to several GnIH group peptides and all NPFF group peptides (Table S1).

### Molecular Characterization of Amphioxus PQRFa Peptide Receptors

**Figure 4:**
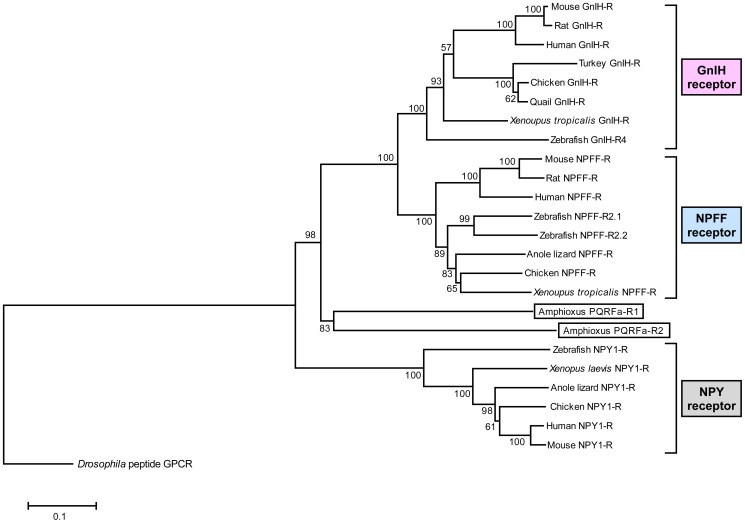
Phylogenetic tree of amphioxus PQRFa peptide receptors, vertebrate GnIH receptors, NPFF receptors and NPY receptors. Scale bar refers to a phylogenetic distance of 0.05 nucleotide substitutions per site. Numbers on the branches indicate bootstrap percentage after 1000 replications in constructing the tree.

In this study, we cloned two putative amphioxus PQRFa peptide receptors named amphioxus PQRFa-R1 and PQRFa-R2, based on the nucleotide sequences of the scaffold BF_V2_167 and BF_V2_95 in the genome database. Analysis of these receptors for regional hydrophobicity revealed seven putative transmembrane domains (TMs; underlined in Figures S5 and S6). The phylogenetic analysis showed that the amphioxus receptors, amphioxus PQRFa-R1 and PQRFa-R2, are closer to GnIH and NPFF receptors compared to neuropeptide Y (NPY) receptors (Figure 4). NPY receptors are included in the phylogenetic tree because these receptors are evolutionarily close to GnIH and NPFF receptors [36].

### Bioactivity of Endogenous Amphioxus PQRFa Peptides

**Figure 5:**
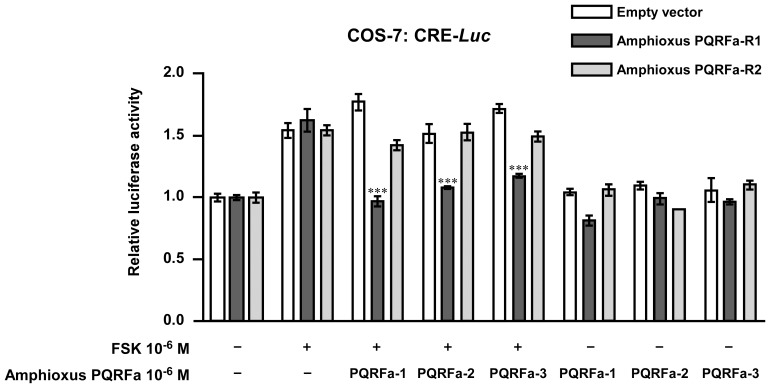
Effect of amphioxus PQRFa peptides on forskolin (FSK)-induced or basal CRE-luciferase activity. COS-7 cells were co-transfected with empty vector (white bars), amphioxus PQRFa-R1-expression vector (dark grey bars), or amphioxus PQRFa-R2- expression vector (light grey bars) along with the CRE-luciferase reporter. Cells were challenged by FSK alone or with amphioxus PQRFa peptides at the concentration of 10^−6^ M for 6 h. The effect of amphioxus PQRFa peptides on basal CRE-luciferase activity was also tested in COS-7 cells transfected with amphioxus PQRFa-R1 or R2.The relative luciferase activity was measured from cell lysates and expressed as fold activation over the respective basal levels. Each column and the vertical line represent the mean ±SEM (*n* = 5–8). ***, *P*<0.001 *vs.* challenged only FSK in each group, one-way ANOVA followed by Turkey's post-tests.

Because GPR147 and GPR74 couple with G_αi_, it was hypothesized that activation of GnIH receptor inhibits adenylate cyclase (AC), thus reduces intracellular cAMP levels and PKA activity (AC/cAMP/PKA pathway) [3], [37]. Indeed, mouse GnIH directly inhibited cAMP production induced by GnRH in mouse gonadotrope cell line (LβT2 cells) [38]. Therefore, we examined the effect of amphioxus PQRFa peptides on AC/cAMP/PKA pathway using a cAMP-response element (CRE) reporter system as described previously [38]. Forskolin (FSK), an AC activator, was used to investigate the inhibitory effect of amphioxus PQRFa peptides on AC/cAMP/PKA pathway through amphioxus receptors (Figure 5). As shown in Figure 5, FSK-induced CRE-luciferase activity was significantly inhibited by amphioxus PQRFa-1, PQRFa-2 and PQRFa-3 at the concentration of 10^−6^ M in COS-7 cells transfected with the amphioxus PQRFa-R1 (Figure 5, dark grey bars). In contrast, all amphioxus PQRFa peptides did not inhibit FSK-induced CRE-luciferase activity in COS-7 cells transfected with the amphioxus PQRFa-R2 (Figure 5, light grey bars). These results suggest that amphioxus PQRFa-R1 is the dominant receptor for amphioxus PQRFa peptides. On the other hand, all amphioxus PQRFa peptides did not modify basal CRE-luciferase activity in COS-7 cells even if they were transfected with the amphioxus PQRFa-R1 (Figure 5).

**Figure 6:**
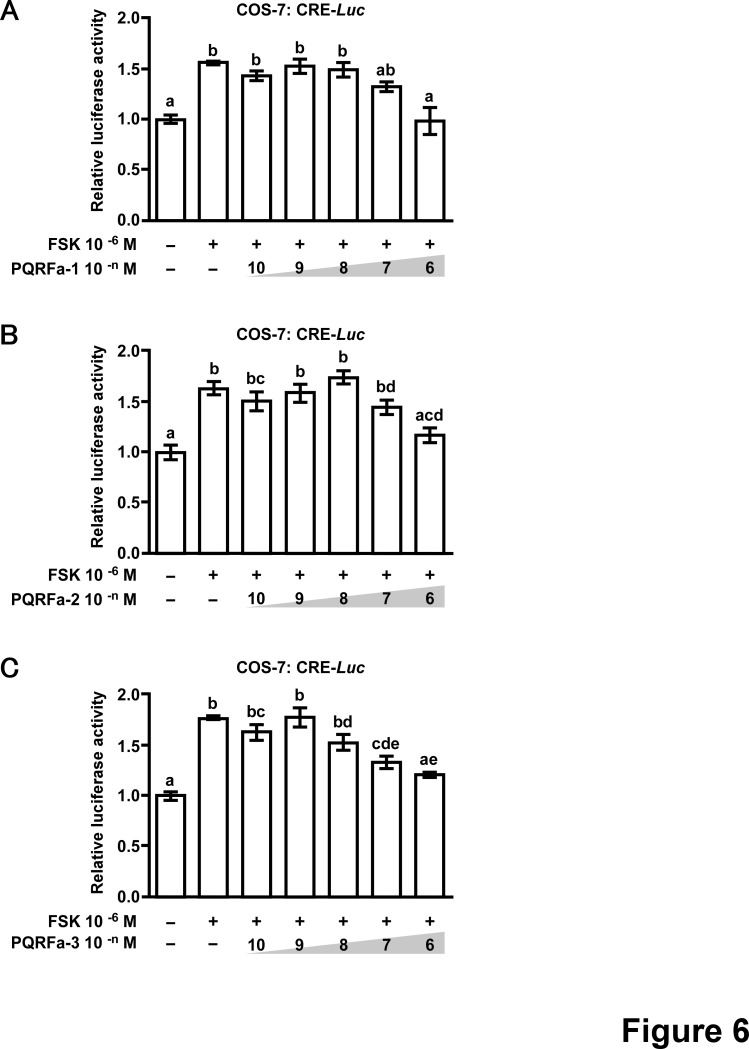
Dose-dependent inhibitory effects of amphioxus PQRFa peptides on forskolin (FSK)-induced CRE-luciferase activity. COS-7 cells were co-transfected with amphioxus PQRFa-R1-expression vector and the CRE-luciferase reporter. Cells were then challenged by FSK and amphioxus PQRFa-1 (*A*), PQRFa-2 (*B*) and PQRFa-3 (*C*) at the concentrations of 10^−10^−10^−6^ M for 6 h. The relative luciferase activity was measured from cell lysates and expressed as fold activation over the respective basal levels. Each column and the vertical line represent the mean ±SEM (*n*=4−6). Letters above the bars indicate significant differences among treatments (bars with the same letter are not significantly different; *P*<0.05; one-way ANOVA followed by Turkey’s multiple comparisons test).

We then investigated the dose dependent effects of amphioxus PQRFa peptides on the inhibition of FSK-induced CRE-luciferase activity in COS-7 cells transfected with the amphioxus PQRFa-R1 (Figure 6). As shown in Figure 6, PQRFa-1 (A) and PQRFa-2 (B) inhibited FSK-induced CRE-luciferase activity at the concentration of 10^−6^ M. On the other hand, PORFa-3 (C) inhibited FSK-induced CRE-luciferase activity at 10^−7^ M and 10^−6^ M, suggesting that amphioxus PORFa-3 is the dominant ligand for the amphioxus PQRFa-R1.

## Discussion

Based on previous studies [23], [24], we hypothesized that GnIH gene and NPFF gene may have diverged from a common ancestral gene through the whole-genome duplication during vertebrate evolution. We have demonstrated that GnIH and NPFF genes are present in the agnathans, the most basal vertebrates [24], [26], [27]. To clarify the evolutionary origin of GnIH gene and NPFF gene, this study therefore investigated their existence in protochordates. We initially searched the genome database of the amphioxus *Branchiostoma floridae*, a most basal chordate, by using blast program to investigate whether amphioxus has similar gene(s) to GnIH gene or NPFF gene. As a result, we found a putative gene encoding three RFa peptides (amphioxus PQRFa peptides) that have the C-terminal PQRFa motif identical to that of vertebrate peptides of the GnIH group and the NPFF group. Subsequently, we cloned a cDNA of amphioxus PQRFa peptide precursor by a combination of 3′/5′ RACE in *Branchiostoma japonicum*. The deduced precursor polypeptide consisted of 208 amino acid residues, including the sequences of three putative amphioxus PQRFa peptides, i.e., amphioxus PQRFa-1, PQRFa-2 and PQRFa-3.

Phylogenetic analysis also showed that amphioxus PQRFa peptide precursor was located before the split of the GnIH group and NPFF group (see Figure 1B), suggesting that amphioxus PQRFa peptide precursor is closely related to the common ancestor of GnIH and NPFF precursors. Synteny analysis suggests that the conserved synteny region exists around the loci of amphioxus PQRFa peptide gene, GnIH gene and NPFF gene although the complete chromosome data are not available in the genome database of *Branchiostoma floridae*. Based on the phylogenetic analysis and synteny analysis, it is suggested that the characteristics of the common ancestral gene of GnIH and NPFF genes are conserved in the amphioxus PQRFa peptide gene. It is interesting that RFa peptides (Sm-npp-13) that possess the C-terminal PQRFa motif were reported by the analysis of EST database of the flatworm *Schistosoma mansoni* that belongs to the phylum Platyhelminthes [39]. The orthologous relationship between the genes of Sm-npp-13, GnIH and NPFF is obscure due to the lack of the synteny data. Further studies are needed to clarify the evolutionary relationship among amphioxus PQRFa peptides, GnIH, NPFF and flatworm PQRFa peptides. As mentioned above, GnIH gene and NPFF gene are located near the *HOXA* and *HOXC* clusters, respectively, suggesting that GnIH and NPFF genes may have duplicated through whole-genome duplications [23], [24]. In primates, prolactin-releasing peptide (PrRP), one of the RFamide peptides, gene is located on the chromosome 2 where *HOXD* cluster is located. However, the location of PrRP gene is distant from *HOXD*, and PrRP does not possess a C-terminal PQRFamide motif that is conserved between GnIH and NPFF. Therefore, we consider that PrRP gene is not a paralogous gene of GnIH or NPFF gene. We could not find other RFamide peptide genes on the *HOXB* and *HOXD* loci. Accordingly, we consider that the paralogous genes of GnIH and NPFF were lost during evolution. The evolutionary relationship among all RFa peptides remains to be clarified.

To investigate the biological action of amphioxus PQRFa peptides, it is essential to identify their receptors. By using genome database search, we found putative amphioxus PQRFa peptide receptors (amphioxus PQRFa-R1 and PQRFa-R2) similar to vertebrate GnIH receptors and NPFF receptors (see Figure 4). Phylogenetic analysis showed that amphioxus PQRFa peptide receptors were closer to vertebrate GnIH and NPFF receptors compared to vertebrate NPY receptors. The phylogenetic positions of the putative amphioxus PQRFa peptide receptors suggest that these receptors are evolutionarily related to vertebrate GnIH and NPFF receptors.

It is important to identify endogenous amphioxus PQRFa peptides for the functional analyses of these ligands. The amphioxus PQRFa peptide sequences are flanked on both ends by the typical endoproteolytic sequences, i.e. KVSR, RSGR, or RFGR on the PQRFa peptide precursor polypeptide sequence (see Figure 1A), suggesting that mature peptides may be generated [40]. Therefore, we performed immunoaffinity purification to identify endogenous amphioxus PQRFa peptides. On the basis of mass spectrometry and post-source decay analysis, the primary structures of the isolated peptides were considered as follows: WDEAWRPQRFa (amphioxus PQRFa-1), GDHTKDGWRPQRFa (amphioxus PQRFa-2) and GRDQGWRPQRFa (amphioxus PQRFa-3). This is the first identification of RFa peptides in protochordates. The C-terminal four amino acids of amphioxus PQRFa peptides were identical to those of several GnIH group peptides and to all NPFF peptides in vertebrates, although the N-terminal sequences of these peptides were diverse (see Table S1).

**Figure 7:**
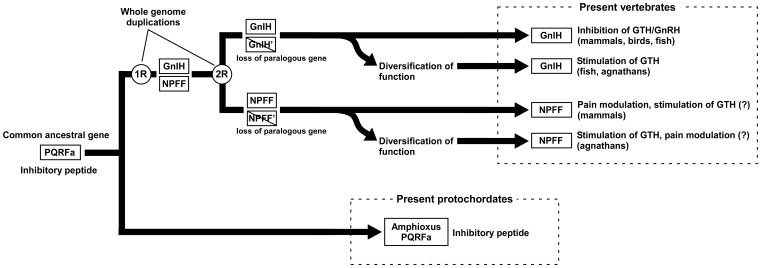
Proposed evolutionary history of GnIH and NPFF genes. GnIH and NPFF genes, originating from a common ancestral PQRFa gene, may have evolved through the whole-genome duplication. The amphioxus PQRFa peptides possess inhibitory action, suggesting that the common ancestor of GnIH and NPFF group peptides was an inhibitory peptide. After the 2 rounds of whole-genome duplications and subsequent loss of paralogous genes, the ancestral gene may have diverged into GnIH gene and NPFF gene. In the present vertebrates, GnIH group peptides inhibit GTH and GnRH in mammals, birds and fish, whereas they stimulate GTH in some fish and agnathans. NPFF group peptides modulate pain transmission in mammals and stimulate GTH in agnathans. It is possible that NPFF group peptides stimulate GTH in mammals and modulate pain transmission in agnathans.

We conducted *in vitro* assays to analyze whether the amphioxus PQRFa peptides have a biological activity. The synthetic amphioxus PQRFa peptides significantly inhibited FSK-induced CRE-luciferase activity in COS-7 cells expressing amphioxus PQRFa-R1. This result suggests that the amphioxus PQRFa-R1 couples with G_αi_ protein and thus amphioxus PQRFa peptides are considered to inhibit AC/cAMP/PKA pathway. Our previous study showed that GnIH directly inhibits cAMP production induced by GnRH in the mouse gonadotrope cell line, LβT2 [38]. Interestingly, NPFF also inhibited FSK-stimulated CRE-luciferase activity [41] or inhibited cAMP levels [42]. The present study suggests that amphioxus PQRFa peptides act as inhibitory peptides on the neuroendocrine system of reproduction and their mode of action is similar to those of vertebrate GnIH and NPFF group peptides. Therefore, the common ancestral peptide of GnIH and NPFF group peptides may have played an inhibitory role in reproduction of the ancestral protochordates. In mammals and birds, numerous studies showed that GnIH acts to inhibit GTH release and synthesis [2], [3]. In fish, zebrafish GnIH (LPXRFa-3) inhibits luteinizing hormone (LH) release in goldfish [20]; whereas goldfish GnIHs (LPXRFa peptides) stimulate the expression of GTH mRNAs in sockeye salmon [43] and grass puffer [21]. Lamprey GnIH (LPXRFa peptide-2) also increased the expression of GTHβ mRNA in the pituitary [24]. Our recent studies further showed that the NPFFs (PQRFa peptides) stimulated the expression of GTHβ mRNA in the pituitary of hagfish [27] and increased the concentration of GnRH-II in the brain of lamprey [44]. Based on the present and previous studies, we hypothesized the evolutionary history of GnIH gene and NPFF gene (Figure 7). The inhibitory action of amphioxus PQRFa peptides suggests that the common ancestor of GnIH and NPFF might have been an inhibitory peptide (Figure 7). After the two rounds of whole-genome duplications and consequent loss of paralogous genes, GnIH and NPFF genes might have diverged from the common ancestral gene. Mammals, birds and some fish have retained the inhibitory role of GnIH in reproduction or NPFF in pain transmission, while some fish and lampreys have diversified the roles of GnIH and NPFF group peptides (Figure 7). The biological function of amphioxus PQRFa peptides is still unclear because GTHs have not been identified in amphioxus. The recent studies identified the genes of glycoprotein hormones (GPA2 and GPB5) in amphioxus that may be the ancestral form of vertebrate glycoprotein hormones including GTHs [45]–[47]. However, the function of these glycoprotein hormones is still unclear. Further studies on the identification of functional GTHs in amphioxus are needed to clarify whether amphioxus PQRFa peptides act as inhibitory peptides on GTH secretion in amphioxus as in mammals, birds and fish.

In conclusion, we identified novel RFa peptides in amphioxus as the first protochordate RFa peptides that have retained the characteristics of the common ancestor of vertebrate GnIH and NPFF group peptides, suggesting that GnIH and NPFF genes have originated from the ancestral PQRFa peptide gene of protochordates. The phylogenetic, synteny and functional analyses indicated that GnIH and NPFF genes have diverged from a common ancestral gene in the ancestral protochordates through whole-genome duplication event during vertebrate evolution (see Figure 7). The role of the common ancestral peptide of reproduction of GnIH and NPFF may have been an inhibitory peptide and the role has retained in mammals, birds and some fish during the course of vertebrate evolution.

## Supporting Information

Figure S1 **Amino acid alignments of the precursors of amphioxus PQRFa peptide, GnIH and NPFF.** Amino acids identical to top sequence are indicated by dots. Gaps marked by hyphens were inserted to optimize homology.
(PDF)

Figure S2 **Amino acid alignments of the receptors of amphioxus PQRFa peptide, GnIH and NPFF.** Amino acids identical to top sequence are indicated by dots. Gaps marked by hyphens were inserted to optimize homology.
(PDF)

Figure S3 **Nucleotide sequence and deduced amino acid sequence of a cDNA encoding amphioxus PQRFa peptides obtained from the genome database of the amphioxus** ***Branchiostoma floridae*** **.** The signal peptide is underlined. The putative amphioxus PQRFa peptides are boxed.
(PDF)

Figure S4 **Chromatogram of MALDI-TOF MS of native amphioxus PQRFa peptides.** (*A*) Molecular ion peaks of 1401.72 m/z ([M+H]^+^) (amphioxus PQRFa-3) and 1598.67 m/z ([M+H]^+^) (amphioxus PQRFa-2) were observed in the fraction 13. (*B*) A molecular ion peak of 1389.97 m/z ([M+H]^+^) (amphioxus PQRFa-1) was observed in the fraction 18.
(PDF)

Figure S5 **Nucleotide sequence and deduced amino acid sequence of a cDNA encoding putative amphioxus PQRFa-R1 of** ***Branchiostoma japonicum*** **.** The putative seven transmembrane domains are indicated by underline and TM.
(PDF)

Figure S6 **Nucleotide sequence and deduced amino acid sequence of a cDNA encoding putative amphioxus PQRFa-R2 of** ***Branchiostoma japonicum*** **.** The putative seven transmembrane domains are indicated by underline and TM.
(PDF)

Table S1 **Comparison of the identified amphioxus PQRFa peptides with previously identified GnIH and NPFF vertebrates.**
(DOC)

Table S2 **Oligonucleotide sequences of primers used for cDNA cloning.**
(DOC)

Table S3 **Genbank accession numbers of the GnIH genes and NPFF genes used for the phylogenetic analysis.**
(DOC)

Table S4 **GenBank accession numbers of the GnIH receptor genes, NPFF receptor genes and NPY receptor 1 genes used for the phylogenetic analysis.**
(DOC)

Materials and Methods S1 **The detailed method for bioinformatics analysis, molecular cloning, DNA sequencing, peptide extraction and immunoaffinity purification, and transient transfection and luciferase assay are provided.**
(DOC)
