# Supplementary material for: Evolutionary Origin of GnIH and NPFF in Chordates: Insights from Novel Amphioxus RFamide Peptides
Source: PLoS One. 2014 Jul 1;9(7):e100962. doi: 10.1371/journal.pone.0100962 (PMC4077772; doi:10.1371/journal.pone.0100962)
Supplement: Figure S1 — Amino acid alignments of the precursors of amphioxus PQRFa peptide, GnIH and NPFF. Amino acids identical to top sequence are indicated by dots. Gaps marked by hyphens were inserted to optimize homology. (PDF) [file pone.0100962.s001.pdf]

```
Human RFRP       : -------------------------------------------------------------------------------MEIISSKL :   8
Primate RFRP     : -------------------------------------------------------------------------------........ :   8
Bovine RFRP      : -------------------------------------------------------------------------------.....L.R :   8
Ovine RFRP       : --------------------------------------------------------------------------------------- :   -
Rat RFRP         : -------------------------------------------------------------------------------.......R :   8
Mouse RFRP       : -------------------------------------------------------------------------------.....L.R :   8
Quail GnIH       : -------------------------------------------------------------------------------......QK :   8
Chicken GnIH     : -------------------------------------------------------------------------------......QK :   8
Sparrow GnIH     : -------------------------------------------------------------------------------.K.....K :   8
Frog GRP         : ---------------------------------------------------------------------------------..F.LC :   6
Zebrafish LPXRFa: -------------------------------------------------------------------------------.SYFALLS :   8
Goldfish LPXRFa  : -------------------------------------------------------------------------------.SYF.LVF :   8
Lamprey LPXRFa   : ------------------------------------------------------------------------MLAGFLL.HC.HFAF :  15
Human NPFF       : --------------------------------------------------------------------------------------- :   -
Bovine NPFF      : --------------------------------------------------------------------------------------- :   -
Rat NPFF         : --------------------------------------------------------------------------------------- :   -
Mouse NPFF       : --------------------------------------------------------------------------------------- :   -
Zebrafish PQRFa  : --------------------------------------------------------------------------------------- :   -
Fugu PQRFa       : --------------------------------------------------------------------------------------- :   -
Lamprey PQRFa    : ---------------------------------------------------------------------MEAKAVSAML.LA.ANC. :  18
Hagfish PQRFa    : --------------------------------------------------------------------------------------- :   -
Amphioxus PQRFa  : ---------------------------------------------------------------------------MRTL.V.TWIST :  12
Squid FMRFa      : MRCWSPCSLFVVIVLHCLSSHSSAAFDLAQACVESQRLSLLPICDTIFAVQDGAQQSSDDGLRSKRFIRFGRALSGDAF.RFGKNVP :  87

Human RFRP       : FILLTLATSSLLTSNIFCADELVISNLHSKE--NYDKYSEPRGYP--KGERSLNFEELKDWGPKNVIKMSTPAVNKMPHSFANLPLR :  91
Primate RFRP     : ................S........S..N..--...........--.R..............................VT..... :  91
Bovine RFRP     : ....M............T..SR.P..Y..K--.........DLGWEK....T.......A..--...NK.V.....P.A...... :  91
Ovine RFRP       : -----------.....T..SR.PS.Y..K--.........DLGWEK....T.........--...N.......P.A...... :  71
Rat RFRP         : .........F....TL.S.....PHF....--G.G..Y.L..I.KGVK....T........A.KD....PAPA......A...... :  93
Mouse RFRP       : .........F....T..T..F..PHF....--GDG....L..I.KGEK....S........A.......PAPA......A...... :  93
Quail GnIH       : .......VAF..PHGA.L....K.S.E...D-.D...Y.T.DSILEEK............S..F...N..T.....N.V...... :  94
Chicken GnIH     : .......VAF..PHGM.L....K.S.E...E-.D...Y.I.DSILEEK............S..F...N..T.....N.V...... :  94
Sparrow GnIH     : ...FA...VVF....SM.L..A.K.R.Q...D-.D..DY.I.DNILEEK........E...S......NPFTAS...N.V...... :  94
Frog GRP         : TF.TLF.F..CFDETKTINL.SQEIYDDLF.SKEDLQNERNGNSDEY.YMG.NSIND.NR.NRLS.QPENEFLQE.SLKPA...... :  93
Zebrafish LPXRFa: LA.GI.S.FM.SEVTALRLPLSGER..NGFTWGQ.SENA.EIPRSLEIQDF...VAPTSGGASSPT.LRLH.I.P.PA.LH...... :  95
Goldfish LPXRFa  : LA.G..S.FM.REVTALRWPLPDD..PDRFTWGQ.LENA.EIPRSLEIEDF...VAPTSGRVSSPT.LRLH.K.T.PT.LH...... :  95
Lamprey LPXRFa   : VAPYPESAHGSPLE.QALQYLTEDDA.LI.KLQSRMNTALEGAMVEEVEDCG.AATDEVQGSRE.RAA.R.GVGQGRSSKTLFQ.Q. : 102
Human NPFF       : ----MDSRQAAALLV.LLLIDGG-CAEGPGGQQE-.QL.AEEDSEPLP------P.DAQTS.SLLHYL.QAMERPGRSQA.LFQ.Q. :  75
Bovine NPFF      : ----MD.RQAAALLL.LLLVTDWSHAEGPGGRDGG.QIFMEEDSGAHP------A.DAQTPRSLLRSL.QAMQRPGRSPA.LFQ.Q. :  77
Rat NPFF         : ----MDSKWAA.LLL.LLLRNWG--HAEEAGSWGE.QVFAEEDKGPHPS.YAHTPDR.QTP.SLMR.L.QAMERP.RNPA.LFQ.Q. :  81
Mouse NPFF       : ----MDSKWAA.LLL.LLLLNWG--HTEEAGSWGE.QVFAGEDKGPHPP.YAHIPDR.QTP.SLFR.L.QAMDTP.RSPA.LFQ.Q. :  81
Zebrafish PQRFa : ----------------------------------MNGLLEDR---------------LLVEMLRSL.HGSQRYERNP.VLHQ.Q. :  36
Fugu PQRFa       : ----MDT.VL.SLLA.VV.MAG.TGA..IQGSP.K.DLPGSEEQ----------------------.AD.LME.RETRN.VLHQ.Q. :  61
Lamprey PQRFa    : L.SAARG.F.S.EEAAMPDSDSS..KDYLA.SVHE.P.RDSFDRASPDAAG.SSS...LLSRLARAFMHFPQRFG.AGP.SLFQ.Q. : 105
Hagfish PQRFa    : --------MDTKVLTALFLLI.SYMAQGATTFDATENDFKEES.EEESWSMGTAPLRGIMERVVRAFSN.PQRFG.ADT.HFFQ.Q. :  79
Amphioxus PQRFa : LFPFL..A.ATG.DPRTTYKVSRWDEAWRP.RFGRSGRGDHTKDGWRPQRFGRGRD.GWRPQRFGRTEAGLRE.LGGEAFPLLQMT. :  99
Squid FMRFa      : D.PFEDKRFLRFGRAAPQL.D..KQA.QRV.SLQRADE.SV.RKRSTDAAPQSSA.GGEQKNDSSAT...KRY..DGED.DVKRF.. : 174

Human RFRP       : FGRNVQE---------ERSAGATANLPLR---SGRNMEVSLVRRVPNLPQRFGRTTTAKSVCRMLSDLCQGSMHSPCANDLFYSMTC : 166
Primate RFRP     : ...TT..---------...T..I.....---..........Q.L.................T.......................... : 166
Bovine RFRP     : .......---------...TR.M.H....---L...R.D..S.W.................T.T....L.Q.....ST.G.L...A. : 166
Ovine RFRP       : .......---------...TRVM.H....R--L...R.D..S.............IA....T.T....L.Q.....ST.G.L----- : 142
Rat RFRP         : ......D---------R..PR.R...EAG-----------T.SHF.S.......-....R.T.T.AG.P.K....LASSE.L.A..R : 159
Mouse RFRP       : ...T.D.---------K..PA.RV..EAG-----------TRSHF.S.......-....-SP.TPA..P.KP...LGSSE.L.V.I. : 158
Quail GnIH       : ...SNP.---------...IKP..Y....---F..AFGE..S..A...SN.S...PL...SIQS.L..P.RFGK.VPIS------LS : 163
Chicken GnIH     : ...SNP.---------...IRP..Y....---F..AFGE..S..A...SY.L...PL...SIQS.L..P.RFGK.VPI.------LS : 163
Sparrow GnIH     : ....YP.---------...IKPFS.....---F..AFGENTPNHS.R.SH.LE..PLV.GSSQS.L..P.RFGK.LAV.------LP : 163
Frog GRP         : ...TSDDKIAKSIPSFD.I.KSIP...Q.FG--RYLSGK.K.QS.A........AQYTNHFVHS.DT.PLRFGR..HS.R.Q.E.NS : 178
Zebrafish LPXRFa: ....A.PGTG------D.APKS.I...Q.FGR.CTMCAR.GTGPSAT.......RNIFALDPLRALA.YTR.PE..SFPKERTQ.HD : 176
Goldfish LPXRFa  : ....T.N-TP------RER.KSNI...Q.FGR.CTMCAR.GTGLSAT.......RNIFPLDPFRALT.YKR.PE..-FPKERTQ.HD : 174
Lamprey LPXRFa   : ...G.PPPAA------DCPES.A.SWAGLQDGNADRASR.EPFWHRTR......RGGDPASPM------------------------ : 159
Human NPFF       : ....T.G---------SWR-NEWLSPRAG-------EG.NSQFWSLAA......--------------------------------- : 112
Bovine NPFF      : ....TRG---------SW.-NKRLSPRAG-------EG..SPFWSLAA......K-------------------------------- : 115
Rat NPFF         : ....AWG---------PW.-KEQLSPQA------------REFWSLAA......K-------------------------------- : 114
Mouse NPFF       : ...SAWG---------SW.-KEQL.PQA------------RQFWSLAA......K-------------------------------- : 114
Zebrafish PQRFa : ...GARS---------GL.TEERIQSRDW-------ET.PGQIWSMA.......K-------------------------------- :  75
Fugu PQRFa       : ....SNG---------PTLLDNEMHPHDW-------EGAPGQIWSMA.......K-------------------------------- : 100
Lamprey PQRFa    : ...GSND--D------.EVPPSLFYRRSWG-------APAEKFW.RA.......KK------------------------------- : 146
Hagfish PQRFa    : ...G.TK--S------DQR.E.GVAERRNS-----QET.PAYVW.RAF.....---------------------------------- : 119
Amphioxus PQRFa : TDLHDDLP-----AMAV.YTPPA.R.RALPLLRLY.RGALSQLINGPPK.PATNREVYPPSL...RAAA..LRGFAHQQ.KDTGE.F : 181
Squid FMRFa      : ...RFMRFGRNQGGVGISADL.NRLGEK.FMRF...P.KRF..FGKSDDK..M.FGRNPEDDLEEEKRFMRFGRGDEF.EEDEEAEA : 261

Human RFRP       : QHQEIQNPDQKQSRRLLFKKIDDAELKQEK-------------------------------------------------- : 196
Primate RFRP     : ...........R......Q...........-------------------------------------------------- : 196
Bovine RFRP     : .P......G..NL..RG.Q...........-------------------------------------------------- : 196
Ovine RFRP       : -------------------------------------------------------------------------------- :   -
Rat RFRP         : ......S.G.E.P..R..TET....R....IGNLQPVLQGAMKL------------------------------------ : 203
Mouse RFRP       : ......S.GG.R...GA.VET....R.P..-------------------------------------------------- : 188
Quail GnIH       : .GV.ESE.GM---------------------------------------------------------------------- : 173
Chicken GnIH     : .GV.ESE.GM---------------------------------------------------------------------- : 173
Sparrow GnIH     : RDI.EFE.GI---------------------------------------------------------------------- : 173
Frog GRP         : HPL..K..E.DSD..KRQAMTFRIRTDL.M-------------------------------------------------- : 208
Zebrafish LPXRFa: YMF.TVED-SE..V.NTDYTAL.--------------------------------------------------------- : 198
Goldfish LPXRFa  : YML.TVEDSVE..V.NKDYT.L.--------------------------------------------------------- : 197
Lamprey LPXRFa   : -------------------------------------------------------------------------------- :   -
Human NPFF       : -------------------------------------------------------------------------------- :   -
Bovine NPFF      : -------------------------------------------------------------------------------- :   -
Rat NPFF         : -------------------------------------------------------------------------------- :   -
Mouse NPFF       : -------------------------------------------------------------------------------- :   -
Zebrafish PQRFa : -------------------------------------------------------------------------------- :   -
Fugu PQRFa       : -------------------------------------------------------------------------------- :   -
Lamprey PQRFa    : -------------------------------------------------------------------------------- :   -
Hagfish PQRFa    : -------------------------------------------------------------------------------- :   -
Amphioxus PQRFa : APPRSN.DWLA.IQ..GL.GRKRRD.S----------------------------------------------------- : 208
Squid FMRFa      : .KRF.RFGRDP.K.F.R.G.SGGEDK.FM.FGRNPEEQEADKRFMRFGRGGAEENELNSEDKRFMRFGRSADKCKGCLEG : 341
```

Figure S1
