# Supplementary material for: Evolutionary Origin of GnIH and NPFF in Chordates: Insights from Novel Amphioxus RFamide Peptides
Source: PLoS One. 2014 Jul 1;9(7):e100962. doi: 10.1371/journal.pone.0100962 (PMC4077772; doi:10.1371/journal.pone.0100962)
Supplement: Figure S2 — Amino acid alignments of the receptors of amphioxus PQRFa peptide, GnIH and NPFF. Amino acids identical to top sequence are indicated by dots. Gaps marked by hyphens were inserted to optimize homology. (PDF) [file pone.0100962.s002.pdf]

```
Human GnIH-R           : ------------------------------MEGEPSQPPNSSWPL------------SQNGTNTEATPATN-----LTFSSYYQHTSPVAAMFIVAYALIFLLC--MVGNTLVCFIVLKNRHMHTVTNMFILNLAVSD--LLVGIF :  95
Mouse GnIH-R           : ------------------------------..A......G...P------------.L.ESDA.TA.VAS-----........S.......A........--................R.............--...... :  95
Rat GnIH-R             : ------------------------------..A......G....------------G...SDV.TSI..S-----........S.......A..V.....--................R.............--...... :  95
QuailGnIH-R            : ------------------------------.QALRH.E.SGDTFN------------GSWDNAS.SQILKEN----Y..LA...S...V...L..TF..M.--.I..I.....V...Q.R..........I.--...... :  96
Chicken GnIH-R         : ------------------------------.QALQHPE.SGDPSN------------GSWANVS.SHLLKEN----Y..LA...S...V...L..TF..M.--.I..I.....V...Q.R..........I.--...... :  96
Turkey GnIH-R          : ------------------------------.QALRHPE.SGDPSN------------GSWANAS.SQVLKEN----Y..LA...S...V...L..TF..M.--.I..I.....V...Q.R..........I.--...... :  96
X. tropicalis GnIH-R   : ----------------------------MA..K.RLERCPLPFNI------------.SP.HGNTTQASRDN----F...L...STSI.....L..TF..M.--.I..M.........Q.R..........I.--...... :  98
Zebrafish GnIH-R4      : ------------------------------------------------------------------------------------------------M.--VFS--------------------------------------- :   5
Human NPFF-R           : ------------------------------.NEKWDTNSSEN.HP------------IW.VND.KHHLYSD---INI.YVN..L.QPQ...I..IS.F...F..--.M...V.....MR.K.......L.....I.--...... :  97
Mouse NPFF-R           : ------------------------------.SEKWDSNSSE..NH------------IWS.ND.QHHWYSD---INI.YVN..L.QPQ...V..SS.L...V..--.....V.....IR........F.....I.--...... :  97
Rat NPFF-R             : ------------------------------.GKRWDSNSSG..DH------------IWS.ND.QHPWYSD---INI.YMN..L.QPH.T.V..SS.F...F..--.....V...V.IR..Y.....F..F...I.--...... :  97
Chicken NPFF-R         : ----------------------------MIKETY.NS-SFN..H------------LLSYNG.YKYLYLE---GNVSYVDF.H.QPS...V..AS.L.....--....GV..YT..RSK..R....L.........--...... :  97
Anole lizard NPFF-R    : ----------------------------MEKKNLDLS.SI.GLH------------MR.CSGYTSACNME---ANVSYVDF.L.QPLI.TI..IS.LF..V..--.M..GV......RSK..R....L.........--...... :  98
X. tropicalis NPFF-R   : ----------------------------M.DEK.DSNLSVI.YR------------KSLN---KTLNYEE---PNI.YVDF.L.RPS...V..AS.L...I..--.L..GV......SSK..R....L.....I.--...... :  95
Zebrafish NPFF-R2.1    : ------------------------------.NESLDLNITLVDDG-----------NWTFI.GSSEFFLP--LNNI.YVG..L.QPS...V...S.L....V.--.I..GV......RSKN.R....L.....I.--...... :  98
Zebrafish NFFF-R2.2    : MDFKDEKNPGYQLVGVSDFHSIVLVAIEEVKASQLKMAVTLLDLNSTGSPPLYSPLENGQNISVNRSTFRPNYYTNI.YVDF.L.EPF.S.I...S.L...IV.--.M..GV......RSKN.R....L.....I.--...... : 142
Human NPY1-R           : ------------------------------MNSTLFSQ-------------------VENHSVHSNFSEKNAQLLAFENDDCHLPLAMIFTLAL..GAVII.G--VS..LALII.I..QKE.RN...IL.V..SF..--...A.M :  92
Mouse NPY1-R           : ------------------------------MNSTLFSK-------------------VENHSIHYNASE-NSPLLAFENDDCHLPLA.IFTLAL..GAVII.G--VS..LALII.I..QKE.RN...IL.V..SF..--...AVM :  91
Chicken NPY1-R         : ------------------------------MNASVLD.------------------LGNNSSHLNFSEKNSQILQFEDEDCHVPLAMVFTLAL..GTVII.G--VS..LALIV.I..QKE.RN...IL.V..SF..--...T.M :  92
Anole lizard NPY-1R    : ------------------------------MNTSLLSL-------------------AGNYS-FQNVSKETSGLAHFQNDDCPVPLVMVFTLAL..GGVI..G--LS..LALII.I..QKE.RN...IL.V..SF..--..IT.M :  91
X. laevis NPY-1R       : ------------------------------MNFSTY--------------------FENLSVPNNISG---NITFPI.EDCALPL.MIFTLAL..GAVII.G--LS..LALII.I..QKE.RN...IL.V..SF..--..AT.M :  87
Zebrafish NPY-1R       : ------------------------------MPDSAFS.PVPPIA-------------ALNCSLDLSNCSITNLSAIAYGDECYGSHSLFVIMA...SAVV..G--VI..LALILVIARQ.EL.N...VL.A..S...--..MAVV :  98
Amphioxus PQRFa-R1     : ------------------------------MDIFNDST.VTNET------------------LPYF----------IEK.K.GAG.MSLI.L..V.V.G..--VA..II..VVII.TPRLR....YL........--...AV. :  81
Amphioxus PQRFa-R2     : ----------------------------MGLEDYLRDK.E.GAN------------------DSWLN.I------PIPK.KQPVYIIII...L.VIV.V..--IL..LV..Y.IARTATLRD..HS.........--....V. :  87
D. melanogaster GPCR   : ----------------------------MDMEYITSSSGNITATTEADFSS----SLGESNV..YNTTEMDANESAGEDEEMLRIAFFIGH.VHQ.YIPV.C.TGSI..I.SV.VFFRTKLRKLSSSFYLAA.....TCF.A.L. : 113


Human GnIH-R           : CMPTTLVDNLITGWPFDNATCKMSGLVQGMSVSASVFTLVAIAVERFRCIVHPFREK--LTLRKALVTIAVIWALALLIMCPSAVTL-TVTREEHH-FMVDARNRSYPLYSCWEAWPEKGMRRVYTTVLFSHIYLAPLALIVVMYA : 237
Mouse GnIH-R           : ..........................................................--.......L.................-........-..L......................K...A...A.............. : 237
Rat GnIH-R             : ..........................................................--.......F.................-........-..L......................K...A...A...V........V : 237
QuailGnIH-R            : ..................M.......................................--...........I..V..........I..-........-..L.TY.N.............TE..KI..........V......I... : 238
Chicken GnIH-R         : ..................M.....................................Q.--...........I..V..........I..-........-...TY.N.............TE...I..........V.......... : 238
Turkey GnIH-R          : .............EQGMGSVSLITAG..QK.C.TTFWKVRSHKPH..........Q.--...........I..V..........I..-........-...TY.N.............TE...I..........V.......... : 238
X. tropicalis GnIH-R   : ..................IM...........................Y...Q.--......II..II..V...I.........-...KDDF.-....DY.NY........E..DTE..KI..............T...I... : 240
Zebrafish GnIH-R4      : --.S--------....AVV.........A.........V..........Y..QQ.--..R.Q.II...F....IA.........-..SQDVL.-....RD.IT...FT......DQS..KI.............VT..AI..T : 137
Human NPFF-R           : ...I..L..I.A...G.TM..I......I..A...........D..Q.V.Y..KP.--..IKT.F.I.MI..V..IT..S....M.-H.QE.KYYRVRLNSQ.KTS.V.W.R.D..NQE..KI......AN......S...I..G : 240
Mouse NPFF-R           : ...I..L..I.A...GSSM..I......I..A...........D....V.Y..KP.--..VKT.F...VI..G..IA..T...IM.-H.QE.KYYRVRLSSH.KTSTV.W.R.D..RHE...I......AT......S...I... : 240
Rat NPFF-R             : ...I..L..I.A...GSSM..I......I..A...........D....V.Y..KP.--..VKT.F.M.VI..G..IT..T...IM.-H.QE.KYYRVRLSSH.KTSTV.W.R.D..NQE...I......AT......S...I... : 240
Chicken NPFF-R         : ......L..I.A...GSMV.....M...I.....I........D......Y..KQ.--..TST.VII.T...I..IA.......M.-Q.QE.K.IRVILGYG.ETR.V.W.R.N..NP...KI......AN......S...I... : 240
Anole lizard NPFF-R    : ......L..I.S...G.IA...N.M...I..............D...S..Y..KQ.--..IWT.IFI.V...V..IA.......M.-K.EE.K.FRVVLGEG.KTS.I.W.R.D..NQD..KI......AN......S...I... : 241
X. tropicalis NPFF-R   : ......L..I.A...GSTV.....M...I..............D......Y..KQ.--..IST.V.I.V...V..IA.......MS-H.KEDKNFRVVIGNS.QTN.I.W.R.D..NPE..KI.......N......S...I... : 238
Zebrafish NPFF-R2.1    : ......L..I......GSMV.....M...I..............D......Y..KQ.--..IST.TFI.VI..V..VS.....G.M.-Q..K.QNIRVFRGN-...S.F.W.R.N..NQE..KI......AN......S...I... : 240
Zebrafish NFFF-R2.2    : .........I......GSLV..L..M...I..............D......Y..KQ.--..ISTSTLI.II..V..IS.....G.M.-Q..K.HRVSILLSDG.TTQ.F.W.R.N..NQE..KI......AN......T...I... : 285
Human NPY1-R           : .L.F.F.YT.MDH.V.GE.M..LNPF..CV.ITV.I.S..L.....HQL.IN.RGWR--PNN.H.Y.G.....V..VASSL.FLIYQ-VM.D.PFQNVT---LDAYKDK.V.FDQF.SDSH.LS...L.LVLQ.FG..CF.FIC.F : 232
Mouse NPY1-R           : .L.F.F.YT.MDH.V.GETM..LNPF..CV.ITV.I.S..L.....HQL.IN.RGWR--PNN.H.YIG.T...V..VASSL.FVIYQ-IL.D.PFQNVS---LAAFKDK.V.FDKF.SDSH.LS...L.LVLQ.FG..CF.FIC.F : 231
Chicken NPY1-R         : .L.F.F.YT.MDH.I.GE.M..LNPF..CA.ITV...S..L..I..HQL.IN.RGWR--PNN.H.YMG..A..V..TASSL.FLIYH-VL.D.PFRNIT---FDEYKDK.V.LDLF.LDTA.LS...T.LVIQ.FG..CF.FIC.L : 232
Anole lizard NPY-1R    : GL.F.F.YT.MDH.V.GE.M..LNPF..CV..TV...S..L..I..HQL.IN.RGWR--PNN.H.YIG..A..II.VFSSL.FLIYH-IL.D.PFTNLT---IEAYKGK.V.MDSF.MESI.LS...A.LVMQ..I..CF.FIC.L : 231
X. laevis NPY-1R       : .L.F..IYT.MDH.I.GEVM..LNEYI.CV..TV.I.S..L..I..HQL.IN.RGWR--PNN.H.CFG.T...GF.MACST.LMMYS-VL.D.PFKNIS---LDSYIGK.V.L.DF..DKF.LS...L..ILQ..G..CF.F.C.T : 227
Zebrafish NPY-1R       : .L.F.FIYTFMDH.V.GAVM..LNS...CC...V.I.S..L..I..HQL.L..RGWR--PS.NH.CLG.SLT....V.TAT.FLLFS-R..DAPLKQLPSVFQEQYRGKVV.V.E..SREIKLT...GMLVLQ.IT..TF.FIC.L : 241
Amphioxus PQRFa-R1     : ...F...EHIL..YQ.GDVM.RVTPMI..V..A.....MT...FD.YKA..F.MK.R--M.I.MMAQIVVG..VSGVA..I.QVFV.-K.V-------TYGPPSGDISVSA.I.I..DTTYKQ...AS...LV.VL..LV.SYF.C : 217
Amphioxus PQRFa-R2     : .I.F...GHIF.ENNLGDVM..V.PML.....AT.....T...FD.YCV....T.DR--..V.Q.VYL.IA..VV.AI..S.QSIVR-RDQ-------SFQ--IGQLT.SV.G.F..SPLL.KA.SAF..VIC.A..VLINTFL.G : 221
D. melanogaster GPCR   : AQWLNFLNVD.YNQNY---F.QFFTFFSYLASFC..WFV..FT....IAVIY.LKRQTMC.V.R.KIVLFCLTLVGC.HCL.YI.IAKP.FMPKLNTTIC.LNSEYKEQLALFNY.DTIVVYA.PF.TIAVLNTCTGCTVWKFATV : 256


Human GnIH-R           : RIARKLCQAPGPAP-------------GGEEAADP--RASRRRARVVHMLVMVALFFTLSWLPLWALLLLIDYGQLSAPQLHLVTVYAFPFAHWLAFFNSSANPIIYGYFNENFRRGFQAAFRARLCPRPSGSHKEAYSERPGG-- : 366
Mouse GnIH-R           : .............R-------------DA...VAEGG............................V........E..EL....LS.....L.......H..........................Q..WL.WAA..Q.......R-- : 368
Rat GnIH-R             : .............R-------------DT...VAEGG.T..........................V........E..EL....LS.....L.......H..........................Q..WP.WAA..Q......NR-- : 368
QuailGnIH-R            : ...F..FKSAA..R-------------STRPEEPEGRKV...K.K.IN..IIM...........T.M..T...H..EH......G.I.............................E....PFWLP.CRQ.RGP.GA.----- : 366
Chicken GnIH-R         : ...F..FKSAA..R-------------.TRPEEPEGRKV...K.K.IN..IIM...........T.M..T..RH..EH......G.V.............................E....PF.LP.RRQ.RGP.GA.----- : 366
Turkey GnIH-R          : ...F..FKSAA..R-------------STRPEEPEGRKV...K.K.IN..IIM...........T.M..T...H..ER......G.I.............................E....PF.LP.RRQ.RGP.GA.----- : 366
X. tropicalis GnIH-R   : ...F..FKSSA.IR-------------.SVSEENEGR.V.K.KV..IY..II.....S.......T.M..T...H..DY..N.IA..V.............V.................E..KVQV.SMKR-E.R.T....KNSGF : 372
Zebrafish GnIH-R4      : ...L..VRS.ASIR-------------DAQ-.E.ESR.VF..KL...N..L....L..V.......I.MM.T...N.TEA..DF.A...V.............V...V...................KMGM.LVEPPQPRRNGTWK----- : 264
Human NPFF-R           : ..GIS.FR.AV.HT--------------.RKNQEQWHVV..KKQKIIK..LI...L.I.......T.MM.S..AD..PNE.QIINI.IY........G...V......F..........E..QLQ..QKRAKPME-..ALKAKS-- : 369
Mouse NPFF-R           : ..GAS.FKTAAHCT--------------.KQRPVQWHVS-KKKQK.IK..LT...L.I.......T.MM.S..TD..PNK.RIINI.IY........C...V......F......N...D..--QI.QKKAKPQE-...L.AKR-- : 366
Rat NPFF-R             : ..GAS.FKTSAHST--------------.KQRLEQWHVS-KKKQK.IK..LT...L.I.......T.MM.S..AD..PNK.RVINI.VY........C...V......F......S...D..--QF.QKKVKPQE-..GL.AKR-- : 366
Chicken NPFF-R         : ..GIA.FNTAM.TV--------------.KHRQEQRHTV.KKKQK.IK..II...L.........T.MM.S..AN..DI..QIINI.IY............I......F.........K...KLQ..SKNIIHRE-V..H.GQS-- : 369
Anole lizard NPFF-R    : ..GIT.FSTSP..C--------------.KQGHER-HSVQKKKLK.IK..II...L.I.......T.AM.S..AD..EN..KIINI.VY............I..V...F...............LQ..SGDGIPRE-I..Q.GPS-- : 369
X. tropicalis NPFF-R   : ..GIT.FKSELNVE--------------NKNHQQ.RHAV...KQK.IK..III..L.I.......T.MM.T..AN.TQN.YQMINI.IY............I......F...............KLQ..SEELERRT-M..H.AQ.-- : 367
Zebrafish NPFF-R2.1    : ..GIT.FKTAM..--------------..KHGH.NRHSV.KKKQ..IK..LI...L.I.......T.MM.T..VK.TEH.YRVINI.IY............V......F............I.KFG...-VG.Q.R-T..H.VQ.-- : 368
Zebrafish NFFF-R2.2    : ..GIT.FKTSM...VSQVGTSGSGSGS.KQCRYESRQLV..KKK..IK..LV...L.I.........MM.S..AS..ER.HRVINI.FY.L......C...V......F......K..K...KLQ..SGH..NRR-T..H.LQ.-- : 428
Human NPY1-R           : K.YIR.KRRNNMMD-----------------KMRDNKYR.SETK.INI..LSIVVA.AVC....TIFNTVF.WNHQII--ATCNHNLL.LLC.LT.MISTCV...F..FL.K..Q.DL.FF.NFCDFRSRDDDYETIAMSTMHT-- : 357
Mouse NPY1-R           : K.YIR.KRRNNMMD-----------------KIRDSKYR.SETK.INI..LSIVVA.AVC....TIFNTVF.WNHQII--ATCNHNLL.LLC.LT.MISTCV...F..FL.K..Q.DL.FF.NFCDFRSRDDDYETIAMSTMHT-- : 356
Chicken NPY1-R         : K.YFR.KKRSNMMD-----------------KMRDSKYR.SETK.INI..ISIVVA.AVC....TIFNIVF.WNHEIL.VATCSHNLL.LIC.LT.MISTCV...F..FL.K..Q.DL.FL.HFCHFRSREEDYETIAMSTMHT-- : 359
Anole lizard NPY-1R    : K.YMR.KRRNNMMD-----------------KMRDNKYR.TETK.INI..ISIVVA.AVC....TIFNTVF.WNHEIL.-ASCSHNLL.LIC.LT.MISTCV...F..FL.K..Q.DL.VF..FCDFHTRDDDYETIAMSTMHT-- : 357
X. laevis NPY-1R       : K.FLR.KRRNNMMD-----------------KIRDNKYR.SETK.INI..LSIVVG.A.C...FFIFN.VF.WNHEAV--ATCNHNLL.LIC.LT.MISTCV...F..FL.K..Q.DL.FF.NFCDFRSREDDYETIAMSTMHT-- : 352
Zebrafish NPY-1R       : K.YTR.QRRNNMME-----------------RIRENKYR.SESK.INI..FSIVVA.AVC....NVFNAV..WNHEV.--MNCTHNLL.SLC.LT.MCSVCI..VF..FL.R..Q.DLR.FRLCKIVSTRENEYDMVAMSTVNT-- : 366
Amphioxus PQRFa-R1     : .VMY..SATSANQS----------------GHRPNQYAV..K.V..LK..IT.VVL.A......YTCWM.DEFAD..LW.RTIISH.I..IG....HS..CV...V..F..S.I.KNLESRDSR.KVGADTRAK.TSPTTATTK-- : 345
Amphioxus PQRFa-R2     : .TGI..WMTKSA.A----------------PK.CS--NQTEQ.S.IIK.M.IIVAV.AIT.M..YSCW..E.F...NNS.SEILHS.IY.V..VM..A..AVD.F...FYSS.L.SRVADIC.LQKTKANVEARNNNNA.KTLD-- : 347
D. melanogaster GPCR   : .RTLTMHKMKPQTN-------------SMPPNSSNSSGGASSAVASYRLSASLKRQKSTGTH.SGQHNVANRQTDDQEQ.QQSQQHQINNCQ.HCEITQKP.RRKVQNSSQLKVTKMLLIVSTVFV.LNLPSCLLRIEAYWETESA : 389


Human GnIH-R           : --LLHRRVFVVVR---------------PSDSGLPSESGPSSGAPRPGRLPLRNGRVAHHGLPREGPG--CSHLPLTIPAWDI------------ : 430
Mouse GnIH-R           : --..R...V.D.Q---------------..............V.G.N..........QDG.....--.N.M......N.------------ : 432
Rat GnIH-R             : --..R...V.D.Q---------------..............V.G............QDG.G...--.N.M......N.------------ : 432
QuailGnIH-R            : -----N...TQPQ---------------A...PPH...R.---------.AS.----------.A.-----------....NG------------ : 399
Chicken GnIH-R         : -----N...TQPQ---------------A...PPH...R.---------.AS.----------.A.-----------V...NG------------ : 399
Turkey GnIH-R          : -----N...TQPQ---------------A...PPH...R.---------.AS.----------.A.-----------....NG------------ : 399
X. tropicalis GnIH-R   : LFGVRN.I..E.Q---------------.....QG.D.CN.TAKG--.MFS..S..I.....VV.DVDNKS.NNTVSV...E.------------ : 438
Zebrafish GnIH-R4      : ----.N....DRD---------------AQGNDRRDFCNG---------------------EQHD--------E.VLEDL.------------- : 297
Human NPFF-R           : -----HVLINTSN---------------QLVQESTFQNPHGE-----TL.YRKSAEKPQQE.VM.ELKETTNSSEI------------------ : 420
Mouse NPFF-R           : -----NI.INTSG---------------LLVQEPV.QNPGGE-----NLGCGKSADNPTQESLI.EM.EATNSTVA------------------ : 417
Rat NPFF-R             : -----NLDINTSG---------------LLVHEPA.QNPSGE-----NLGCRKSADNPTQESLM.ET.EATNSTET------------------ : 417
Chicken NPFF-R         : -----NAILPAAN-------------YQTP-QDQACQNVKAE----RKPIRKG.WMNNQQD.MM.DLEEPYNNR-----MK------------- : 422
Anole lizard NPFF-R    : -----NAILPAAA-------------PQTP-.HHLAPTVAAG----SAP--KGSWMDNTQV.MM.SLEKT.NNNGVKQDMT------------- : 425
X. tropicalis NPFF-R   : -----NAILPAAS-------------HNTARQNSAF.KQS.D----DKESDK.KPLTSDQD.IM.DLEKFSNNNGIQKDMV------------- : 426
Zebrafish NPFF-R2.1    : -----NS.QP.NL-------------QPSTEPIS-LN.LEN-------NSSR.MNHINEQD.VM.DLEKVSEYSMEGASL-------------- : 422
Zebrafish NFFF-R2.2    : -----NA.LPANLPLTKQTALETGAPRPGQGLIQGLV.VSNGKGLGERSQSSGKENMKEQD.IM.DLEKV-MYDL------------------ : 497
Human NPY1-R           : -------DVSKTS----------------LKQAS.VAFKKINNNDDNEKI-------------------------------------------- : 384
Mouse NPY1-R           : -------DVSKTS----------------LKQAS.VAFKKI.MND-NEKV-------------------------------------------- : 382
Chicken NPY1-R         : -------DVSKTS----------------LKQAS.VAFKKINSDD-DDKI-------------------------------------------- : 385
Anole lizard NPY-1R    : -------DISKTS----------------LKQAS.ITFKKI.DSD-DEKI-------------------------------------------- : 383
X. laevis NPY-1R       : -------DVSKTS----------------LKQAS.IA------------------------------------------------------ : 366
Zebrafish NPY-1R       : -------DVSKMS----------------LKMSSLDL-------------------------------------------------------- : 380
Amphioxus PQRFa-R1     : -------QTNIR.----------------DVIE.RPLN---AIWTGGS.GSRADYKDHDGDYKDHDIDYKDDDDK------------------ : 394
Amphioxus PQRFa-R2     : -------IVRKP.----------------.FQVNLPDNRNMPEVANES.GSRADYKDHDGDYKDHDIDYKDDDDK------------------ : 399
D. melanogaster GPCR   : R----NQNSTIALQYIFHAFFITNFGINFVLYCVSGQNFRKAVLSIFR.VSSAQREAGNTQVTVSEYCRNTGTSTRRRMMTQHCWNEMHELHPLK : 480
```

Figure S2
