# Supplementary material for: Evolutionary Origin of GnIH and NPFF in Chordates: Insights from Novel Amphioxus RFamide Peptides
Source: PLoS One. 2014 Jul 1;9(7):e100962. doi: 10.1371/journal.pone.0100962 (PMC4077772; doi:10.1371/journal.pone.0100962)
Supplement: Figure S3 — Nucleotide sequence and deduced amino acid sequence of a cDNA encoding amphioxus PQRFa peptides obtained from the genome database of the amphioxus Branchiostoma floridae . The signal peptide is underlined. The putative amphioxus PQRFa peptides are boxed. (PDF) [file pone.0100962.s003.pdf]

```
ATGCGCACGTTAGTGGTGTTGACTTGGATATCTACGATCTTCCCGCTCCTGCTGGCCGCT  60
 M  R  T  L  V  V  L  T  W  I  S  T  I  F  P  L  L  L  A  A    20
                  Signal peptide
CCACAGGCGGACCCCCGCACCACCTACAAAGTCTCTCGGTGGGATGAGGCCTGGCGCCCG 120
 P  Q  A  D  P  R  T  T  Y  K  V  S  R [W  D  E  A  W  R  P    40
                                        amphioxus PQRFa-1
CAGCGGTTCGGCCGGAGCGGTCGGGGAGACACCAAGGACGGATGGCGACCACAACGCTTC 180
 Q  R  F] G  R  S  G  R [G  D  T  K  D  G  W  R  P  Q  R  F]   60
                                amphioxus PQRFa-2
GGTAGAGGGCGCTACGAGCAGGGCTGGCGGCCGCAGAGGTTCGGACGGAACGAGGGGCTG 240
 G  R [G  R  Y  E  Q  G  W  R  P  Q  R  F] G  R  N  E  G  L    80
          amphioxus PQRFa-3
GCGGGCCTGCGAGAGGTCCTGGACGGCGAGGCCTTCCCGCTGCTGCAGATGACCAGAACC 300
 A  G  L  R  E  V  L  D  G  E  A  F  P  L  L  Q  M  T  R  T   100
GACCTCCGCCATGACCTGCCCGGCATCGGTTACACACCGGGAACCGCACACGGCAGTCTG 360
 D  L  R  H  D  L  P  G  I  G  Y  T  P  G  T  A  H  G  S  L   120
CGGGCTCTACCTCTCCTCCGTCTGTACGACCGCGGTGCACTTCAGCTTATCAACGGAGCC 420
 R  A  L  P  L  L  R  L  Y  D  R  G  A  L  Q  L  I  N  G  A   140
GCCAAACAGTCCTCCACAAACCCGCCTTCACTCAGAATGATCCGGGCAGCGGCCGAGAGT 480
 A  K  Q  S  S  T  N  P  P  S  L  R  M  I  R  A  A  A  E  S   160
CTTCAGGGATTTGCGCGCGGAGGCGATCAACAGGATGAGGAGGAGATGTTCGCGCCTCCA 540
 L  Q  G  F  A  R  G  G  D  Q  Q  D  E  E  E  M  F  A  P  P   180
CGAAGCACCGACGACTGGCTCGCCGAGATCCAGAGGCTGGGACTCCGCGGAAGGAAGCGA 600
 R  S  T  D  D  W  L  A  E  I  Q  R  L  G  L  R  G  R  K  R   200
CGGGATGTCAGCTAG                                              612
 R  D  V  S  *                                                 204
```

Figure S3
