# Supplementary figures and images for: Evolutionary Origin of GnIH and NPFF in Chordates: Insights from Novel Amphioxus RFamide Peptides
Source: PLoS One. 2014 Jul 1;9(7):e100962. doi: 10.1371/journal.pone.0100962 (PMC4077772; doi:10.1371/journal.pone.0100962)

**A (Fraction 13)**

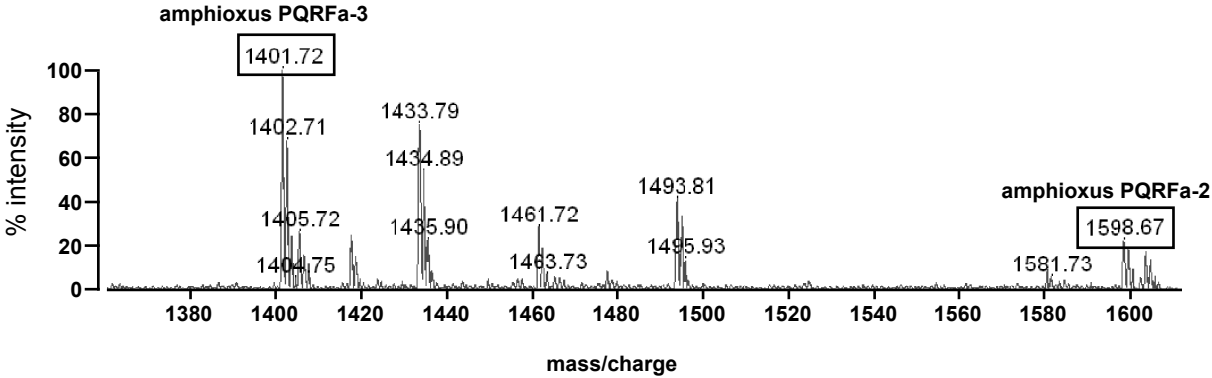

**B (Fraction 18)**

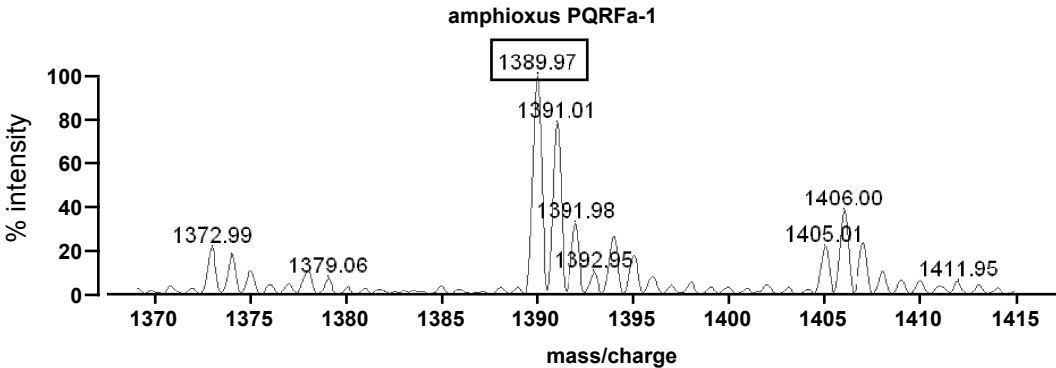

**Figure S4**

Supplement: Figure S4 — Chromatogram of MALDI-TOF MS of native amphioxus PQRFa peptides. (A) Molecular ion peaks of 1401.72 m/z ([M+H]+) (amphioxus PQRFa-3) and 1598.67 m/z ([M+H]+) (amphioxus PQRFa-2) were observed in the fraction 13. (B) A molecular ion peak of 1389.97 m/z ([M+H]+) (amphioxus PQRFa-1) was observed in the fraction 18. (PDF) [file pone.0100962.s004.pdf]
