# Supplementary material for: Evolutionary Origin of GnIH and NPFF in Chordates: Insights from Novel Amphioxus RFamide Peptides
Source: PLoS One. 2014 Jul 1;9(7):e100962. doi: 10.1371/journal.pone.0100962 (PMC4077772; doi:10.1371/journal.pone.0100962)
Supplement: Figure S5 — Nucleotide sequence and deduced amino acid sequence of a cDNA encoding putative amphioxus PQRFa-R1 of Branchiostoma japonicum . The putative seven transmembrane domains are indicated by underline and TM. (PDF) [file pone.0100962.s005.pdf]

```
ATGGACATCTTCAACGACAGCACCAACGTCACAAACGAGACCCTTCCGTACTTCATAGAG   60
 M  D  I  F  N  D  S  T  N  V  T  N  E  T  L  P  Y  F  I  E    20
AAGTACAAGCACGGGGCTGGTGTCATGTCCCTCATCATCTTAGCATATGTCCTTGTCTTC  120
 K  Y  K  H  G  A  G  V  M  S  L  I  I  L  A  Y  V  L  V  F    40
GGGCTGTGCGTAGCTGGAAACATCATCGTCTGCGTGGTGATTATCAAAACCCCGAGGCTC  180
 G  L  C  V  A  G  N  I  I TM1 V  C  V  V  I  I  K  T  P  R  L    60
AGGACTGTAACGAATTACTTGATCCTGAACTTGGCCGTGAGTGATCTGCTAGTGGCCGTG  240
 R  T  V  T  N  Y  L  I  L  N  L  A  V  S  D  L  L  V  A  V    80
TTCTGCATGCCTTTCACTCTGGTTGAGCACATTCTGACAGGCTACCAGTTTGGGGACGTC  300
 F  C  M  P  F  T  L  V TM2 E  H  I  L  T  G  Y  Q  F  G  D  V   100
ATGTGCCGAGTGACGCCCATGATCCAGGGTGTGTCCGTGGCGGCATCAGTGTTCACAATG  360
 M  C  R  V  T  P  M  I  Q  G  V  S  V  A  A  S  V  F  T  M   120
ACGGCCATCGCGTTTGACAGGTACAAGGCGATCGTGTTCCCGATGAAAGAGCGGATGACT  420
 T  A  I  A  F  D TM3 R  Y  K  A  I  V  F  P  M  K  E  R  M  T   140
ATCCGGATGATGGCGCAGATCGTGGTGGGAATCTGGGTGAGCGGCGTCGCCATCATGATC  480
 I  R  M  M  A  Q  I  V  V  G  I  W  V  S  G  V  A  I  M  I   160
CCCCAGGTCTTCGTTCTAAAGGTTGTAACGTACGGGCCGCCAAGTGGCGACATCTCAGTC  540
 P  Q  V  F  V  L  K  V  V  T TM4 Y  G  P  P  S  G  D  I  S  V   180
TCGGCGTGTATTGAGATCTGGCCTGACACTACCTACAAACAGGTTTACACTGCCTCCTTG  600
 S  A  C  I  E  I  W  P  D  T  T  Y  K  Q  V  Y  T  A  S  L   200
TTCTCACTTGTGTATGTCCTCCCATTGTTGGTCATTTCATACTTCTATTGCCGTGTGATG  660
 F  S  L  V  Y  V  L  P  L  L  V  I  S  Y  F  Y TM5 C  R  V  M   220
TACAAACTGTCAGCAACGTCTGCAAATCAGAGTGGGCATCGTCCCAACCAGTACGCCGTC  720
 Y  K  L  S  A  T  S  A  N  Q  S  G  H  R  P  N  Q  Y  A  V   240
TCGCGGAAACGGGTCAGGGTTCTAAAGATGTTGATCACTGTTGTGGTGCTGTTCGCCCTG  780
 S  R  K  R  V  R  V  L  K  M  L  I  T  V  V  V  L  F  A  L   260
TCATGGCTACCCCTGTACACATGTTGGATGCTTGACGAATTCGCTGATCTGTCTCTGTGG  840
 S  W  L  P  L  Y  T  C  W  M  L TM6 D  E  F  A  D  L  S  L  W   280
CAACGAACGATCATAAGCCATTACATCTTTCCCATTGGCCACTGGCTCGCCCACTCCAAC  900
 Q  R  T  I  I  S  H  Y  I  F  P  I  G  H  W  L  A  H  S  N   300
AGCTGTGTCAACCCCATCGTCTATGGCTTCTTCAATTCCAACATTCGAAAAAACCTTGAG  960
 S  C  V  N  P  I  V  Y  G  F  F TM7 N  S  N  I  R  K  N  L  E   320
AGCAGGGATTCGAGGAGAAAAGTAGGGGCAGACACTAGGGCAAAGAAAACATCGCCGACG 1020
 S  R  D  S  R  R  K  V  G  A  D  T  R  A  K  K  T  S  P  T   340
ACAGCCACTACAAAGCAAACGAATATCAGGAGAGACGTAATCGAGCTGCGGCCTCTTAAC 1080
 T  A  T  T  K  Q  T  N  I  R  R  D  V  I  E  L  R  P  L  N   360
GCTATCTGGACCGGGGGCTAA                                        1101
 A  I  W  T  G  G  *                                            366
```

Figure S5
