# Supplementary material for: Evolutionary Origin of GnIH and NPFF in Chordates: Insights from Novel Amphioxus RFamide Peptides
Source: PLoS One. 2014 Jul 1;9(7):e100962. doi: 10.1371/journal.pone.0100962 (PMC4077772; doi:10.1371/journal.pone.0100962)
Supplement: Figure S6 — Nucleotide sequence and deduced amino acid sequence of a cDNA encoding putative amphioxus PQRFa-R2 of Branchiostoma japonicum . The putative seven transmembrane domains are indicated by underline and TM. (PDF) [file pone.0100962.s006.pdf]

```
ATGGGCTTGGAAGATTACCTCCGCGACAAGAATGAAAGCGGCGCAAATGACTCTTGGTTA   60
 M  G  L  E  D  Y  L  R  D  K  N  E  S  G  A  N  D  S  W  L   20
AACAACATTCCAATCCCCAAGTACAAGCAGCCAGTGTACATCATCATCATCTTTATAGTG  120
 N  N  I  P  I  P  K  Y  K  Q  P  V  Y  I  I  I  I  F  I  V   40
CTGTATGTTATTGTGTTCGTGCTGTGCATTTTGGGTAATCTCGTGGTGTGTTATATCATC  180
 L  Y  V  I  V  F  V  L  C  I  L TM1 G  N  L  V  V  C  Y  I  I   60
GCGAGAACTGCAACACTGCGAGATGTTACCCACTCCTTCATCTTGAACCTTGCCGTAAGT  240
 A  R  T  A  T  L  R  D  V  T  H  S  F  I  L  N  L  A  V  S   80
GACCTCCTAGTGGGGGTCTTCTGCATCCCGTTCACCTTGGTGGGCCACATTTTTACCGAG  300
 D  L  L  V  G  V  F  C  I  P  F TM2 T  L  V  G  H  I  F  T  E  100
AACAACCTTGGTGACGTCATGTGTAAAGTGAGTCCTATGCTGCAAGGCATGTCGGTAGCC  360
 N  N  L  G  D  V  M  C  K  V  S  P  M  L  Q  G  M  S  V  A  120
ACATCGGTGTTCACCCTGACTGCCATCGCATTTGACAGATACTGCCTGATTGTCCACCCG  420
 T  S  V  F  T  L  T  A  I  A TM3 F  D  R  Y  C  L  I  V  H  P  140
ACACGTGACCGCCTGACCGTCCGCCAAGCAGTGTATCTGATCATCGCCATTCTGGTTGTT  480
 T  R  D  R  L  T  V  R  Q  A  V  Y  L  I  I  A  I  L  V  V  160
GCTGCCATTATCATGTCACCACAGTCTATTGTTCGCCGTGACCAGTCCTTCCAGATTGGC  540
 A  A  I  I  M  S  P  Q  S  I  V TM4 R  R  D  Q  S  F  Q  I  G  180
CAGCTGACCCTGTCTGTGTGTGGAGAGTTCTGGCCGTCTCCGCTGCTGCGTAAGGCCTAC  600
 Q  L  T  L  S  V  C  G  E  F  W  P  S  P  L  L  R  K  A  Y  200
AGCGCCTTCCTGTTCGTCATCCGTTACGCAGCACCCGTTCTCATCAACACCTTCCCGTAC  660
 S  A  F  L  F  V  I  R  Y  A  A  P  V  L  I  N  T  F  P  Y TM5  220
GGCAGAACAGGCATCAAGCTCTGGATGAGCAAGTCAGCTGCTGCACCTAAAGCCTGCAGT  720
 G  R  T  G  I  K  L  W  M  S  K  S  A  A  A  P  K  A  C  S  240
AACCAGACTGAGCAACGAAGCCGCATCATCAAGATGATGGTGATCATCGTCGCCGTATTT  780
 N  Q  T  E  Q  R  S  R  I  I  K  M  M  V  I  I  V  A  V  F  260
GCCATTACCTGGATGCCGCTCTACAGCTGTTGGCTATTAGAGGACTTCGGTCAGCTGAAC  840
 A  I  T  W  M  P  L  Y  S  C  W  L  L TM6 E  D  F  G  Q  L  N  280
AACAGCCAATCAGAGATCCTGCACAGCTACATCTACCCGGTAGCTCGCGTGATGGCGTTT  900
 N  S  Q  S  E  I  L  H  S  Y  I  Y  P  V  A  R  V  M  A  F  300
GCCAACAGCGCGGTGGATCCGTTCATCTACGGCTTCTACAGCAGCAACCTGCGCAGTCGC  960
 A  N  S  A  V  D  P  F  I  Y  G  F  Y TM7 S  S  N  L  R  S  R  320
GTTGCAGACATCTGCCGCCTACAGAAGACAAAGACCAACGTGGAAGCTCGTAACAACAAC 1020
 V  A  D  I  C  R  L  Q  K  T  K  T  N  V  E  A  R  N  N  N  340
AATGCTGAGAAGACGCTGGACATTGTCCGCAAGCCACGGTCCTTCCAGGTGAACCTCCCA 1080
 N  A  E  K  T  L  D  I  V  R  K  P  R  S  F  Q  V  N  L  P  360
GACAACAGAAACATGCCCGAAGTAGCCAACGAATAG                         1113
 D  N  R  N  M  P  E  V  A  N  E  *                             371
```

Figure S6
