# Supplementary material for: Evolutionary Origin of GnIH and NPFF in Chordates: Insights from Novel Amphioxus RFamide Peptides
Source: PLoS One. 2014 Jul 1;9(7):e100962. doi: 10.1371/journal.pone.0100962 (PMC4077772; doi:10.1371/journal.pone.0100962)
Supplement: Table S1 — Comparison of the identified amphioxus PQRFa peptides with previously identified GnIH and NPFF vertebrates. (DOC) [file pone.0100962.s007.doc]

**Table S1.** Comparison of the identified amphioxus PQRFa peptides with previously identified GnIH and NPFF

in vertebrates

Sequence

Name

Reference

Animal

WDEAWRPQRFamide

GDHTKDGWRPQRFamide

GRDQGWRPQRFamide

WDEAWRPQRFamide

GDTKDGWRPQRFamide

GRYEQGWRPQRFamide

**PQRFa-1**

**PQRFa-2**

**PQRFa-3**

**PQRFa-1***

**PQRFa-2***

**PQRFa-3***

**This study**

**This study**

**This study**

**This study**

**This study**

**This study**

*B. japonicum*

*B. japonicum*

*B. japonicum*

*B. floridae*

*B. floridae*

*B. floridae*

SGVGQGRSSKTLFQPQRFamide

AALRSGVGQGRSSKTLFQPQRFamide

SEPFWHRTRPQRFamide

SGTGLSATLPQRFamide

DGVQGGDHVPNLNPNMPQRFamide

SLKPAANLPLRFamide

SIPNLPQRFamide

SVPNLPQRFamide

SIKPSAYLPLRFamide

SSIQSLLNLPQRFamide

SIKPFANLPLRFamide

SIKPFSNLPLRFamide

ANMEAGTMSHFPSLPQRFamide

VPNLPQRFamide

LPXRFa-1a

LPXRFa-1b

LPXRFa-2

LPXRFa-3

LPXRFa-2*

GRP/R-RFa

GRP-RP-1

LPXRFa-1

GnIH

GnIH-RP-2

GnIH

GnIH

RFRP-3

RFRP-3

Lamprey

Lamprey

Lamprey

Goldfish

Grass puffer

Frog

Frog

Newt

Quail

Quail

Starling

Zebrafinch

Rat

Human

(24)

(24)

(24)

(14)

(21)

(10) (12)

(11)

(13)

(1)

(15)

(18)

(17)

(6)

(8)

**GnIH**

**group**

SWGAPAEKFWMRAMPQRFamide

AFMHFPQRFamide

AGPSSLFQPQRFamide

NPSVLHQPQRFamide

DWETVPGQIWSMAVPQRFamide

FLFQPQRFamide

SLAAPQRFamide

AGEGLNSQFWSLAAPQRFamide

PQRFa

PQRFa-RP-1

PQRFa-RP-2

PQRF-1*

PQRF-2*

NPFF

NPSF

NPAF

Lamprey

Lamprey

Lamprey

Zebrafish

Zebrafish

Bovine

Human

Human

(26)

(26)

(26)

(48)

(48)

(49)

(50)

(50)

**NPFF group**

The C-terminal conserved motifs are shaded in red and grey. The C-terminal five amino acids are boxed in

the dotted line. *Putative sequences.

**References**

1. Oehlmann VD, Korte H, Sterner C, Korsching SI (2002) A neuropeptide FF-related gene is expressed selectively in neurons of the terminal nerve in Danio rerio. Mech Dev 117: 357–361.
2. Bonnard E, Burlet-Schiltz O, Francés B, Mazarguil H, Monsarrat B, et al. (2001) Identification of neuropeptide FF-related peptides in rodent spinal cord. Peptides 22: 1085–1092.
3. Burlet-Schiltz O, Mazarguil H, Sol JC, Chaynes P, Monsarrat B, et al. (2002) Identification of neuropeptide FF-related peptides in human cerebrospinal fluid by mass spectrometry. FEBS Lett 532: 313–318.
