# Supplementary material for: Evolutionary Origin of GnIH and NPFF in Chordates: Insights from Novel Amphioxus RFamide Peptides
Source: PLoS One. 2014 Jul 1;9(7):e100962. doi: 10.1371/journal.pone.0100962 (PMC4077772; doi:10.1371/journal.pone.0100962)
Supplement: Table S2 — Oligonucleotide sequences of primers used for cDNA cloning. (DOC) [file pone.0100962.s008.doc]

**Table S2. Oligonucleotide sequences of primers used for cDNA cloning**

Name Sequence

For PQRFa peptide precursor cDNA

GSP-1 5'-GACACCAAGGACGGATGGCG-3'

GSP-2 5'-TAGCTGACATCCCGTCGCTTCCTTC-3'

GSP-3 5'-CCACAACGCTTCGGTAGAGG-3'

GSP-4 5'-TCCTCCTCATCCTGTTGATC-3'

GSP-5 5’-GAACCGACCTCCATGATGAC-3’

GSP-6 5'-CATGGCCGTCAGATACACAC-3'

GSP-7 5'-ACAGCCGGAGGAGAGGTAGA-3

GSP-8 5'-GCAGCCGGCGGTGTGTATCT-3'

GSP-9 5'-TGGAGGTCGGTTCTGGTCAT-3'

For PQRFa-R1 cDNA

DG1 5'-CTTCATCCTBAACYTGGCCGTSAG-3'

DG2 5'-AGYGAYCTGCTRGTGGSCATHTTYTG-3'

DG3 5'-ACGCWGSWGTTGGARWASGCSAGCCA-3'

DG4 5'-TTGAAGWAGCCRTAGATRATGGGATTG-3'

GSP-10 5'-TCTGGGTGAGCGGCGTCGCCATC-3'

GSP-11 5'-GGGCCGCCAAGTGGCGACATCTCAG-5'

GSP-12 5'-GCCGAGACTGAGATGTCGCCACTTGG-5'

GSP-13 5'-CGAGACGGCGTACTGGTTGGGACG-5'

For PQRFa-R2 cDNA

DG5 5'-TTCATCTTGAACCTKGCCGTSAGYGACCT-3'

DG6 5'-TCTGTGTTCACMCTGACBGCCATCGCT-3'

DG7 5'-GGATCCACCGYGCTGTTGGVGAACGCCAT-3'

DG8 5'-CCGTAGATGAASGGATCCACCGYGCTGTT-3'

GSP-14 5'-GTGTGTGGAGAGTTCTGGCCGTC-3'

GSP-15 5'-GCGTAAGGCCTACAGCGCCTTCC-3'

GSP-16 5'-TGATGCGGCTTCGTTGCTCAGTCTGGT-3'

GSP-17 5'-ACGGCGACGATGATCACCATCATCTTG-3'
