# Supplementary material for: Evolutionary Origin of GnIH and NPFF in Chordates: Insights from Novel Amphioxus RFamide Peptides
Source: PLoS One. 2014 Jul 1;9(7):e100962. doi: 10.1371/journal.pone.0100962 (PMC4077772; doi:10.1371/journal.pone.0100962)
Supplement: Table S3 — Genbank accession numbers of the GnIH genes and NPFF genes used for the phylogenetic analysis. (DOC) [file pone.0100962.s009.doc]

**Table S3. Genbank accession numbers of the GnIH genes and NPFF genes used for the phylogenetic analysis**

Name Accession numbers

Human RFRP AB040290

Primate RFRP EU834942

Bovine RFRP AB040291

Ovine RFRP NM_001127268

Rat RFRP AB040288.1

Mouse RFRP AB040289

Quail GnIH AB039815

Chicken GnIH AB193126

Sparrow GnIH AB128164

Frog GRP AB080743

Goldfish LPXRFa AB078976

Zebrafish LPXRFa AB193139

Lamprey LPXRFa AB661773

Human NPFF NM_003717

Bovine NPFF AF148699

Rat NPFF AF148700

Mouse NPFF AF148701

Fugu PQRFa AB193134

Zebrafish PQRFa AB193140

Lamprey PQRFa AB233469

Hagfish PQRFa AB639150

Squid FMRFa FJ896403
