# Supplementary material for: Evolutionary Origin of GnIH and NPFF in Chordates: Insights from Novel Amphioxus RFamide Peptides
Source: PLoS One. 2014 Jul 1;9(7):e100962. doi: 10.1371/journal.pone.0100962 (PMC4077772; doi:10.1371/journal.pone.0100962)
Supplement: Table S4 — GenBank accession numbers of the GnIH receptor genes, NPFF receptor genes and NPY receptor 1 genes used for the phylogenetic analysis. (DOC) [file pone.0100962.s010.doc]

**Table S4. GenBank accession numbers of the GnIH receptor genes, NPFF receptor genes and NPY receptor 1 genes used for the phylogenetic analysis**

Name Accession number

Human GnIH-R NM_022146

Mouse GnIH-R NM_001177511

Rat GnIH-R NM_022291

Quail GnIH-R AB183891

Chicken GnIH-R NM_204362

Turkey GnIH-R ENSMGAT00000019193

*Xenopus tropicalis* GnIH-R XM_002935965

Zebrafish GnIH-R4 ENSDARG00000091788

Human NPFF-R AF268899

Mouse NPFF-R NM_133192

Rat NPFFR2 NM_023980

Chicken NPFF-R NM_001034825

Anole lizard NPFF-R XM_003221835

*Xenopus tropicalis* NPFF-R XM_002940351

Zebrafish NPFF-R2.1 NM_001105109

Zebrafish NPFF-R2 .2 XP_690069

Human NPY1-R NM_000909

Mouse NPY1-R NM_010934

Chicken NPY1-R NM_001031535

Anole lizard NPY1-R XM_003221700

*Xenopus laevis* NPY1-R NM_001085879

Zebrafish NPY1-R NM_001102391

*D. melanogaster* peptide GPCR AY217746.1
