# Supplementary material for: Evolutionary Origin of GnIH and NPFF in Chordates: Insights from Novel Amphioxus RFamide Peptides
Source: PLoS One. 2014 Jul 1;9(7):e100962. doi: 10.1371/journal.pone.0100962 (PMC4077772; doi:10.1371/journal.pone.0100962)
Supplement: Materials and Methods S1 — The detailed method for bioinformatics analysis, molecular cloning, DNA sequencing, peptide extraction and immunoaffinity purification, and transient transfection and luciferase assay are provided. (DOC) [file pone.0100962.s011.doc]

**Supporting data**

**Supporting Materials and Methods**

**Bioinformatics analyses**

The Joint Genome Institute (JGI) genome database of the amphioxus *Branchiostoma floridae* (http://genome.jgi-psf.org/Brafl1/Brafl1.home.html) was used to search for a ortholog of GnIH or NPFF precursor gene or its receptor gene using BLASTP or TBLASTN program using lamprey PQRFa precursor protein (AB661773) or *Xenopus* GnIH receptor (XM_002935965 ) as queries. As a result, scaffold Bf_V2_187 for amphioxus PQRFa peptide precursor gene, scaffold BF_V2_167 for amphioxus PQRFa-R1 and scaffold BF_V2_95 for amphioxus PQRFa-R2 appeared. We then analyzed the nucleotide sequences by GENSCAN [33] (http://genes.mit.edu/GENSCAN.html) to obtain putative amphioxus PQRFa peptide precursor gene or its receptor genes. We also performed the genome synteny analysis by comparing the gene loci of amphioxus PQRFa peptide, GnIH and NPFF, or their receptor genes in amphioxus, human, anole lizard and zebrafish by using JGI genome database and Ensembl Genome Browser (http://www.ensembl.org/index.html).

**Molecular cloning**

Based on the nucleotide sequences of the scaffold Bf_V2_187 for amphioxus PQRFa peptide precursor gene, scaffold BF_V2_167 for amphioxus PQRFa receptor 1 (PQRFa-R1) and scaffold BF_V2_95 for amphioxus PQRFa receptor 2 (PQRFa-R2) in the genome database of *Branchiostoma floridae* (http://genome.jgi-psf.org/Brafl1/Brafl1.home.html), we performed molecular cloning of the cDNAs using the tissues of *Branchiostoma japonicum*. Total RNA was extracted from the head and body of amphioxus including nerve cord or gonad using ISOGEN (NIPPON GENE, Tokyo, Japan) in accordance with the manufacturer’s instructions. All PCR amplifications were performed in a reaction mixture containing Bio *Taq* polymerase (Bioline, London, UK), rTaq polymerase (Takara Bio Inc., Shiga, Japan) or Prime Star HS DNA polymerase (Takara Bio Inc.), 0.2 mM dNTP, and 0.5% dimethyl sulfoxide using a thermal cycler (Program Temp Control SystemPC-700; ASTEC, Fukuoka, Japan). All primers used in this study are summarized in Table S2. For the amphioxus PQRFa peptide cDNA, first-strand cDNA was synthesized with the oligo(dT)-anchor primer supplied in the 5’/3’ rapid amplification of cDNA ends (RACE) kit (Roche Diagnostics, Basel, Switzerland). We first amplified partial cDNA sequence of amphioxus PQRFa with the gene-specific primer (GSP)-1 corresponding to the nucleotide position 4367432 to 4367451 in the scaffold Bf_V2_187 and GSP-2 complementary to the nucleotide position 4367874 to 4367898 in the scaffold Bf_V2_187. The first round PCR products were further amplified with GSP-3 corresponding to the nucleotide position 4367453 to 4367472 in the scaffold Bf_V2_187 and GSP-4 complementary to the nucleotide position 4367789 to 4367808 in the scaffold Bf_V2_187. All PCRs consisted of 30 cycles of 30 s at 94 °C, 30 s at 55 °C and 1 min at 72 °C (10 min for the last cycle). After the determination of the partial cDNA sequence, we performed 3’/5’RACE to obtain the full length of amphioxus PQRFa peptide precursor cDNA. For 3’ RACE, the first-round PCR products were amplified with the anchor primer (Roche Diagnostics) and GSP-5 corresponding to the nucleotide position 304 to 323 in Figure 1A. The first round PCR products were further amplified with the anchor primer (Roche Diagnostics) and GSP-6 corresponding to the nucleotide position 332 to 351 in Figure 1A. All PCRs consisted of 30 cycles of 30 s at 94 °C, 30 s at 55 °C and 30 s at 72 °C (10 min for the last cycle). For 5’ RACE, template cDNA was synthesized with GSP-7, complementary to nucleotides 374 to 393 in Figure 1A. This cDNA synthesis was followed by dA-tailing of the cDNA with dATP and terminal transferase (Roche Diagnostics). The poly(A) tailed cDNA was amplified with the oligo(dT)-anchor primer and GSP-8, complementary to nucleotides 342 to 361 in Figure 1A. It was followed by further amplification of the first-round PCR products with the anchor primer and GSP-9, complementary to nucleotides 297 to 316 in Figure 1A. Both the first and second-round PCRs were performed as described above.

For the amphioxus PQRFa-R1 and PQRFa-R2 cDNAs, the first-strand cDNA was synthesized using SMARTer RACE cDNA Amplification Kit (Takara Bio Inc.). The partial cDNA sequence of amphioxus PQRFa-R1 was amplified with the degenerate primer (DG)-1 and DG-4. The first round PCR products were further amplified with DG-2 and DG-3.

PCRs consisted of 30 cycles of 30 s at 94 °C, 15 s at 53 °C and 1 min at 72 °C. After the determination of the partial cDNA sequence, we performed 3’/5’RACE to obtain the full length of amphioxus PQRFa-R1 cDNA as described above. GSP-10 and GSP-13 were used for the first round PCR. GSP-11 and GSP-12 were used for the second round PCR. The first round and second round PCRs consisted of 30 cycles of 30 s at 94 °C, 15 s at 63 °C and 2 min at 72 °C. The partial cDNA sequence of amphioxus PQRFa-R2 was amplified with the degenerate primer (DG)-5 and DG-8. The first round PCR products were further amplified with DG-6 and DG-7. PCRs consisted of 30 cycles of 30 s at 94 °C, 15 s at 58 °C and 1 min at 72 °C. After the determination of the partial cDNA sequence, we performed 3’/5’RACE to obtain the full length of amphioxus PQRFa-R2 cDNA as described above. GSP-14 and GSP-17 were used for the first round PCR. GSP-15 and GSP-16 were used for the second round PCR. The first round PCRs consisted of 30 cycles of 30 s at 94 °C, 15 s at 58 °C and 2 min at 72 °C. The second round PCRs consisted of 30 cycles of 30 s at 94 °C, 15 s at 60 °C and 2 min at 72 °C. All second-round PCR products were subcloned into a pGEM-T Easy vector in accordance with the manufacturer’s instructions (Promega, Madison, WI). The DNA inserts of the positive clones were amplified by PCR with universal M13 primers.

**DNA sequencing**

All nucleotide sequences were determined with a BigDye Terminator v3.1 Cycle Sequencing Kit (Applied Biosystems, Foster City, CA), and a model 3130 Genetic Analyzer (Applied Biosystems). Universal M13 primers or gene-specific primers were used to sequence both strands. All nucleotide sequences were analyzed by using DNA Sequencing Analysis Software v5.1 (Applied Biosystems). SignalP 3.0 (http://www.cbs.dtu.dk/services/SignalP/) was used to identify the signal peptide sequence.

**Peptide extraction and immunoaffinity purification.** 250 adult amphioxus were collected, immediately frozen on dry ice, and stored at –80 °C until used. The tissues were boiled and homogenized in 5% acetic acid as described previously [24,26,27]. The homogenate was centrifuged at 10,000 × *g* for 30 min at 4 °C, and the resulting precipitate was again homogenized and centrifuged. The two supernatants were pooled and concentrated by using a rotary evaporator at 40 °C. After precipitation with 75% acetone, the supernatant was passed through a disposable C18 cartridge column (Sep-Pak Vac; Waters, Milford, MA), and the retained material eluted with 60% methanol was loaded onto an immunoaffinity column. The affinity chromatography was performed as described previously [26,27]. The antiserum against PQRFa peptide [26] was conjugated to Protein A Sepharose 4B (Amersham Pharmacia Biotech, Uppsala, Sweden) as an affinity ligand. The tissue extract was applied to the column at 4 °C, and the adsorbed materials were eluted with 0.3 M acetic acid containing 0.1% 2-mercaptoethanol. An aliquot of each fraction (1 ml) was analyzed by a dot immunoblot assay with the antiserum against PQRFa peptide according to our previous methods [24,26,27].

**Transient transfection and luciferase assay.** Amphioxus PQRFa peptides used for luciferase assay were synthesized using a peptide synthesizer (PSSM-8; Shimadzu). COS-7 cells were maintained in Dulbecco’s modified Eagle’s medium (DMEM; GIBCO/Invitrogen, Carlsbad, CA) supplemented with high glucose (4.5 g/L) containing 10% fetal bovine serum (GIBCO/Invitrogen) and 1% penicillin/streptomycin antibiotics (GIBCO/Invitrogen) in a humidified 5% CO_2_ atmosphere at 37 °C. For luciferase assay, COS-7 cells were plated in 24-well plates and grown to 70–80% confluence for 24 h before transfection. Cells were then co-transfected with 200 ng of p3XFLAG-CMV-14 expression vector (Sigma-Aldrich Co., St Louis, MO) containing the amphioxus PQRFa-R1, PQRFa-R2, or empty vector, 200 ng of the pGL4.29[luc2P/CRE/Hygro] (firefly luciferase reporter construct; Promega, Madison, WI) and 5 ng of pRL-null (renilla luciferase reporter construct; Promega) using FuGENE HD Transfection Reagent (Promega) according to the manufacturer’s instructions. Cells were starved overnight in serum-free DMEM and then challenged by forskolin (FSK) (Santa Cruz Biotechnology, Santa Cruz, CA) and amphioxus PQRFa peptides (PQRFa-1, -2 and -3) for 6 h. Cell extracts were prepared, and luciferase activity was measured using the dual-luciferase reporter assay system (Promega). The ratio of firefly luciferase activity to renilla luciferase activity was used as the results to coordinate the differences in transfection efficiency among samples. All assays were performed in duplicate and repeated three times. Statistical significance was assessed by Prism statistical software (GraphPad Software Inc., La Jolla, CA).
